# Synthesis and Biological Evaluation of Isomeric Artemisinin Trimers as Novel Antitumor Agents

**DOI:** 10.3390/molecules31081228

**Published:** 2026-04-08

**Authors:** Zejin Zhang, Along Li, Bingying Jiang, Typhaine Bejoma, Yongxi Zhao, Fujiang Guo, Yajuan Li, Huiyu Li, Qingjie Zhao

**Affiliations:** 1 State Key Laboratory of Discovery and Utilization of Functional Components in Traditional Chinese Medicine, Shanghai Frontiers Science Center for TCM Chemical Biology, Innovation Research Institute of Traditional Chinese Medicine, Shanghai University of Traditional Chinese Medicine, Shanghai 201203, China; 22023629@shutcm.edu.cn (Z.Z.); educationpharma5@gmail.com (T.B.); 62023047@shutcm.edu.cn (Y.Z.); 2 Institute of Interdisciplinary Integrative Medicine Research, Shanghai University of Traditional Chinese Medicine, Shanghai 201203, China; lialong615@sina.com; 3 Shandong Fushun Biotechnology Co., Ltd., No. 39 Keji Avenue, High-Tech Zone, Yantai 264035, China; 4 School of Pharmacy, Shanghai University of Traditional Chinese Medicine, Shanghai 201203, China; 13856783145@163.com (B.J.); gfj@shutcm.edu.cn (F.G.); 5 College of Mathematics and Physics, Shanghai University of Electric Power, Shanghai 201306, China

**Keywords:** artemisinin derivatives, trimer, chiral configuration, antitumor activity, breast cancer

## Abstract

While artemisinin and its derivatives demonstrate broad antitumor potential, the stereochemical influence on the bioactivity of higher-order artemisinin assemblies remains inadequately characterized. Herein, we report the synthesis, chromatographic separation, and structural elucidation of four stereoisomeric artemisinin trimers, followed by systematic evaluation of their antitumor efficacy against MCF-7 and MDA-MB-231 breast cancer cell lines. All trimers exhibited potent cytotoxicity against MCF-7 cells (IC_50_ < 0.09 μM), with trimer **6a** (*β*, *β*, *β*) demonstrating robust antitumor activity in both in vitro and in vivo xenograft models. Remarkably, pronounced stereochemistry-dependent activity emerged against MDA-MB-231 cells: **6a** displayed approximately 100-fold greater potency than **6b** (*β*, *β*, *α*) and 6.6-fold superiority over gemcitabine. Mechanistic investigations revealed that **6a** downregulates Cyclin D1, CDK4, and CDK6 expression, thereby inducing G0/G1 phase cell cycle arrest. These findings underscore the pivotal role of stereochemical configuration in modulating artemisinin trimer bioactivity and provide rational guidance for structure-based design of artemisinin-derived anticancer therapeutics.

## 1. Introduction

Artemisinin and its derivatives, like dihydroartemisinin and artemether, are widely utilized as antimalarial agents. Recent studies have revealed their considerable potential for inhibiting various tumors [1,2,3,4,5]. Their favorable safety profile and low cost make them promising candidates for adjuvant anticancer therapy [6,7,8]. Mechanistically, these compounds modulate key signaling pathways, including suppression of pro-cancer cascades such as Wnt/β-catenin and PI3K/AKT/mTOR, to inhibit tumor growth [6,9,10,11]. In antitumor therapy, they exhibit synergistic effects integrated with chemotherapeutic agents, such as cisplatin and sorafenib [12,13,14,15,16]. Furthermore, artemisinin effectively inhibits tumor growth in mouse models of breast cancer (BC) xenografts [17,18,19,20].

**Figure 1:**
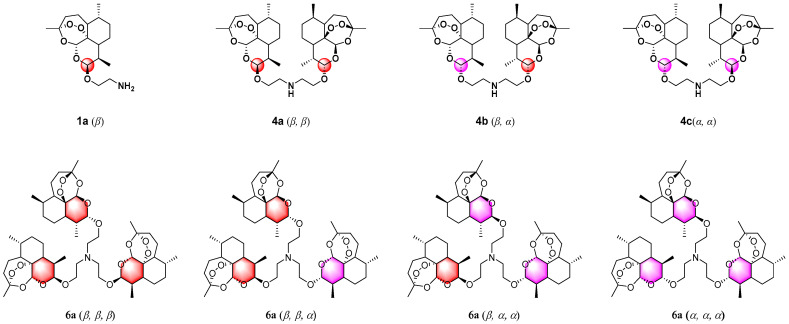
Structures of **1a**, **4a**, **4b**, **4c**, **6a**, **6b**, **6c** and **6d**. Compounds **1a**, **4a**, and **6a** were previously reported in our previous work [29].

In recent years, numerous studies have demonstrated that trimeric compounds exhibit potent antitumor activity [21,22,23]. By designing trimers (or polymers) to enhance antitumor efficacy, robust data and mechanistic explanations have been provided [24,25]. The stereochemistry at the C-10 position of artemisinin derivatives is a critical structural feature, defined by the orientation of the substituent relative to the artemisinin scaffold. According to the conventions established in the literature [26,27], an α-configuration is assigned when the C-10 hydrogen (H-10) is axial, typically characterized by a large coupling constant (J ≈ 9–10 Hz) with the adjacent H-9 in the ^1^H NMR spectrum. Conversely, a β-configuration is assigned when H-10 is equatorial, indicated by a small coupling constant (J ≈ 3–4 Hz). Moreover, our prior research indicates that the anti-breast cancer efficacy of artemisinin dimers with the *β*-configuration outperforms that with the *α*-configuration [28]. Specifically, the (*β*, *β*) dimer outperforms the (*β*, *α*) and (*α*, *α*) configurations. Based on the **4a** (*β*, *β*) configuration, we have synthesized **6a** (*β*, *β*, *β*) and discovered that the artemisinin trimer **6a** (*β*, *β*, *β*) exhibits greater anti-breast cancer effectiveness than the dimer **4a** (*β*, *β*) and the monomer **1a** (*β*) (Figure 1) in cell line MCF-7. Furthermore, we also identified that it exerts its anti-breast cancer activity by activating the apoptosis and iron depletion pathways [29]. However, the other three configurations of the artemisinin trimers ((*β*, *β*, *α*), (*α*, *α*, *α*), (*β*, *α*, *α*)) have not yet been synthesized, and the differences in their anti-tumour activity remain unknown.

To compare the anti-breast cancer activity of different configurational isomers, we synthesized and comprehensively characterized three previously unreported artemisinin trimers **6b**, **6c**, and **6d**, and evaluated the anti-tumor activity of four configurational trimers (**6a**, **6b**, **6c**, **6d**) against two BC cell lines (MCF-7 and MDA-MB-231). Compounds that showed good activity in both cell lines were selected for further studies on their in vivo anti-breast cancer activity and preliminary exploration of their mechanism of action.

## 2. Results and Discussion

### 2.1. Synthesis of Artemisinin Trimers **6a** (β, β, β), **6b** (β, β, α), **6c** (β, α, α) and **6d** (α, α, α)

**Scheme 1:**
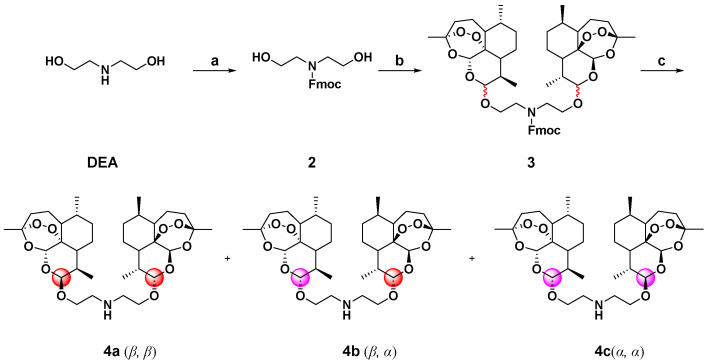
Synthesis of Artemisinin Dimers **4a**, **4b**, and **4c** configurations. Reagents and Conditions: (a) Et3N, Fmoc-Cl, DCM, room temperature, 2 h; (b) BF_3_·Et_2_O, DHA, DCM; (c) piperidine DMF, room temperature, 1 h. Compounds **2**, **3**, and **4a** were previously reported in our previous work [29].

First, our research synthesized the isomeric artemisinin dimers **4a** (*β*, *β*), **4b** (*β*, *α*), and **4c** (*α*, *α*). As shown in Scheme 1, the synthesis commenced with commercially available diethanolamine, and the amino group was preserved with Fmoc-Cl at 0 °C. Subsequent reaction with DHA afforded intermediate **2**, which was then utilized to generate the desired isomeric compounds **3**, incorporating protective group strategies. After column chromatography purification, we obtained the corresponding intermediate compounds as white solids, **4a** with a yield of 28%, **4b** with a yield of 22%, and **4c** with a yield of 5% (Scheme 1).

**Scheme 2:**
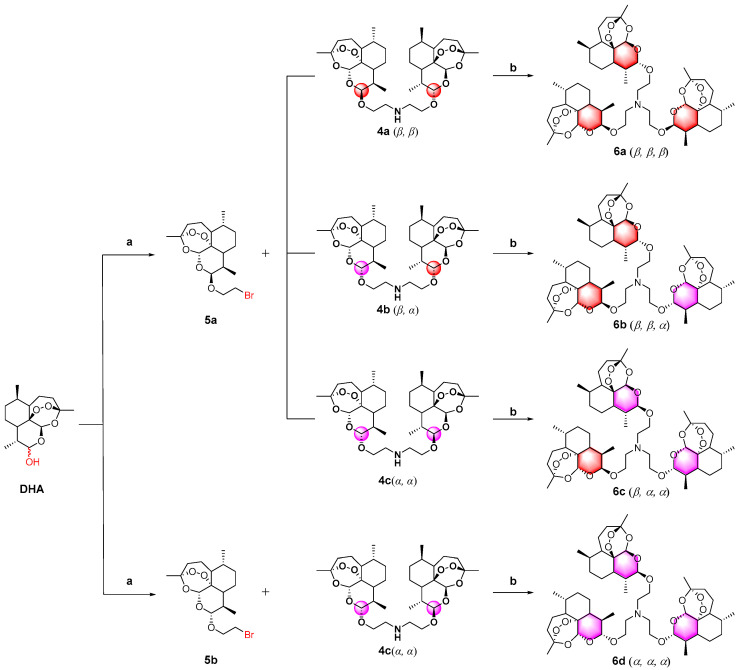
Synthesis Routes for Artemisinin Trimers **6a**, **6b**, **6c** and **6d** configurations. Reagents and Conditions: (a) 2-Bromoethanol, BF_3_·Et_2_O, DCM, room temperature, 2 h. (b) **4a**/**4b**/**4c**, potassium carbonate, N, N-dimethylformamide, 40 °C, 48 h. Compounds **4a**, **5a**, and **6a** were previously reported in our previous work [29].

Next, isomeric artemisinin trimers, namely **6a**, **6b**, **6c** and **6d**, were synthesized as depicted in Scheme 2. Compounds **5a** and **5b** were prepared via a catalytic reaction utilizing DHA and 2-bromoethanol. These components were co-dissolved in dichloromethane and treated with boron trifluoride etherate (BF_3_·Et_2_O) as a catalyst. Subsequently, compound **5a** reacts with artemisinin dimer **4a** to yield the target compound **6a** as a white solid in the 80% yield. Compound **5a** reacts with artemisinin dimer **4b** to yield the target compound **6b** as a white solid in 61% yield. Compound **5a** reacted with artemisinin dimer **4c** to yield the target compound **6c** as a white solid in the 61% yield. Compound **5b** reacted with artemisinin dimer **4c** to yield the target compound **6d** as a white solid in the 52.0% yield. In the relevant molecular framework, the β-orientation positions the substituent (or leaving group) in a sterically less hindered environment, which facilitates attack by bulky nucleophiles such as substituted diethanolamines. Consequently, the fully β-configured trimer **6a** exhibited the highest yield in the S_N_2 reaction. In contrast, the α-orientation introduces 1,3-diaxial interactions with the artemisinin skeleton, causing the reaction center to become sterically buried and less accessible. This geometry is highly unfavorable for S_N_2 reactions, which require a linear trajectory for nucleophilic attack, thereby resulting in the lowest yield for the fully α-configured trimer **6d**. As the number of α-configured centers increases progressively from **6a** to **6d**, steric hindrance accumulates, leading to a stepwise decrease in yield. The comparable yields of **6b** and **6c** are attributed to their similar steric profiles, likely due to the presence of a common sterically sensitive site in both mixed isomers.

### 2.2. H NMR and ^13^C NMR of Compounds **6a** (β, β, β), **6b** (β, β, α), **6c** (β, α, α) and **6d** (α, α, α)

**Figure 2:**
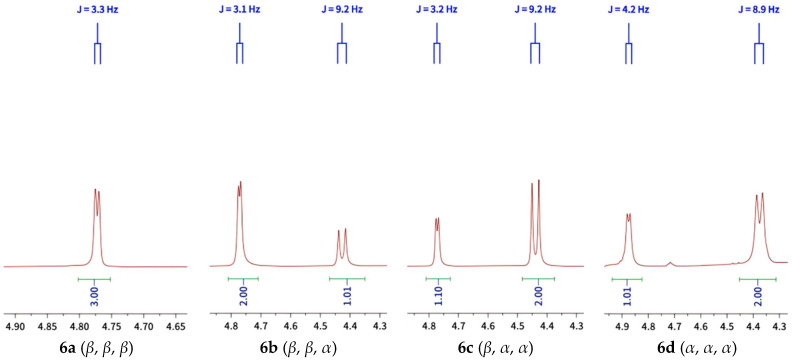
H-10 signal analysis of the (*β*, *β*, *β*)-configuration, (*β*, *β*, *α*)-configuration, (*β*, *α*, *α*)-configuration, and (*α*, *α*, *α*)-configuration.

The structures of the artemisinin trimers **6a**, **6b**, **6c** and **6d** were established via 1D and 2D NMR spectroscopy. The ^1^H NMR and ^13^C NMR signals were assigned utilizing HMQC and HMBC methods, as indicated in Table 1. Figure 2 displays partial ^1^H NMR spectra of compounds **6a**, **6b**, **6c** and **6d** at 400, 500 or 600 MHz, including chemical shifts and coupling constants to H-9 and H-10. Specifically, in **6a**, H-10/10′/10″ at *δ* = 4.76 possessed a constant of coupling with H-9/9′/9″ of *J* = 3.3 Hz. In **6b**, H-10/10′ at *δ* = 4.75 possessed a constant of coupling with H-9/9′ of *J* = 3.1 Hz, and H-10″ at *δ* = 4.41 had a constant of coupling with H-9″ of *J* = 9.2 Hz. In **6c**, H-10 at *δ* = 4.74 had a constant of coupling with H-9 of *J* = 3.2 Hz, H-10′/10″ at *δ* = 4.41 had a constant of coupling with H-9′/9″ of *J* = 9.2 Hz. In compound **6d**, H-10 at *δ* = 4.94 had a constant of coupling with H-9 of *J* = 4.2 Hz, H-10′/10″ at *δ* = 4.44 had a constant of coupling with H-9′/9″ of *J* = 8.9 Hz. These data reveal subtle structural distinctions among the four compounds.

### 2.3. Structural Diagrams of Compounds **6a** (β, β, β), **6b** (β, β, α), **6c** (β, α, α) and **6d** (α, α, α)

**Figure 3:**
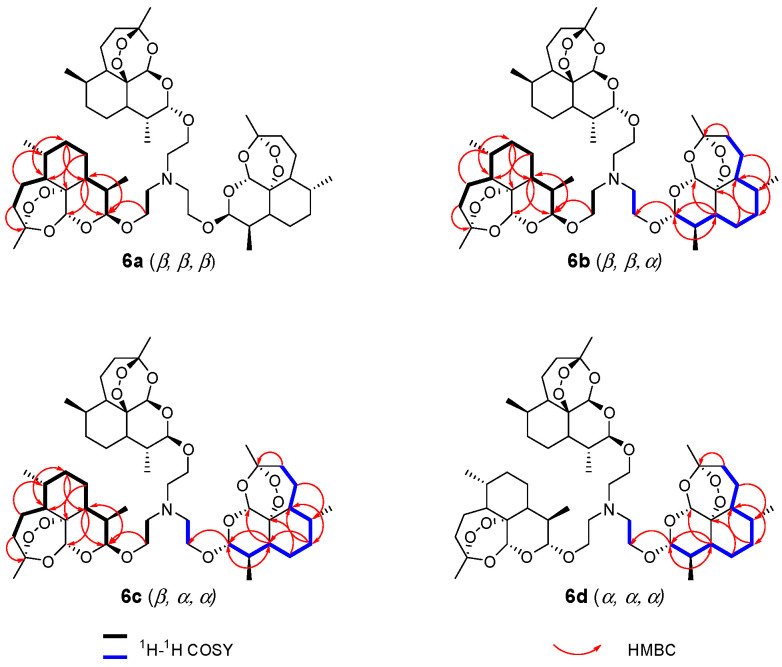
^1^H-^1^H COSY and HMBC data for compounds **6a**, **6b**, **6c** and **6d** (Supporting Information pages S8–S13). Compound **6a** was previously reported in our previous work [29].

**Figure 4:**
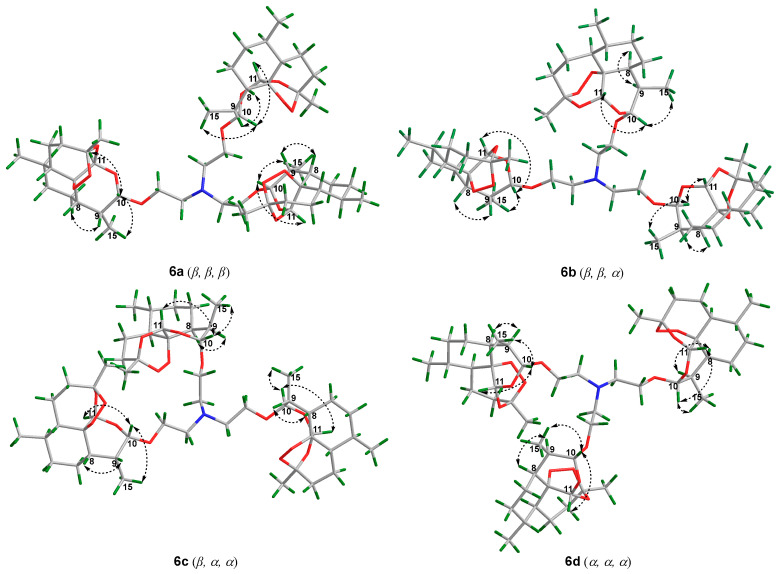
^1^H-^1^H NOESY correlations for compounds **6a**, **6b**, **6c** and **6d** (Supporting Information pages S9, S11 and S13). Compound **6a** was previously reported in our previous work [29].

The molecular framework of compounds **6a**, **6b**, **6c** and **6d** was determined using ^1^H-^1^H COSY and HMBC spectra. The data revealed that compounds **6a**, **6b**, and **6c** have the same *β*-oriented two-dimensional structure, and compounds **6b**, **6c**, and **6d** have the same *α*-oriented two-dimensional structure, as shown in Figure 3. Additionally, NOESY correlations were analyzed to assess the space orientation of the artemisinin trimers. In compound **6a**, the correlation between δ 4.76 (3H, d, H-10/10′/10″) and δ 2.64–2.59 (3H, m, H-9/9′/9″) demonstrated the *β*-orientation of H-10/10′/10″. In compound **6b**, the correlation between δ 4.76 (3H, d, H-10/10′) and δ 2.64–2.59 (3H, m, H-9/9′) confirms the *β*-orientation of H-10/10′, while the correlation between δ 4.76 (3H, d, 10″) and δ 2.64–2.59 (3H, m, 9″) confirms the *α*-orientation of H-10″. In compound **6c**, the correlation between δ 4.76 (3H, d, H-10) and δ 2.64–2.59 (3H, m, H-9) confirms the *β*-orientation of H-10, while the correlation between δ 4.76 (3H, d, 10′/10″) and δ 2.64–2.59 (3H, m, 9′/9″) confirms the *α*-orientation of H-10′/10″. In compound **6d**, the correlation between δ 4.76 (3H, d, H-10/10′/10″) and δ 2.64–2.59 (3H, m, H-9/9′/9″) confirms the *α*-orientation of H-10/10′/10″ (as shown in Figure 4). (Supporting Information pages S8–S13).

### 2.4. Antitumor Effects of **6a** (β, β, β), **6b** (β, β, α), **6c** (β, α, α) and **6d** (α, α, α) in the MCF-7 Model

The cytotoxic effects of artemisinin dimer **4a** (*β*, *β*) and trimer **6a** (*β*, *β*, *β*) were assessed against MCF-7 BC cells. The trimer **6a** exhibited significantly greater potency, with an IC_50_ value of 0.09 ± 0.03 μM, compared with 3.14 ± 0.54 μM for dimer **4a**. Furthermore, the activity of **6a** surpassed that of the reference compounds cisplatin (IC_50_ = 2.41 ± 0.27 μM) and gemcitabine (IC_50_ = 4.33 ± 0.36 μM) [29]. Building on these previous findings, we continued to investigate the differences in antitumor effects among of the four distinct configurations for artemisinin trimers. As shown in Table 2, the in vitro cytotoxicity of **6a**, **6b**, **6c**, **6d** and **4a**, **4b**, **4c** was evaluated and compared in breast cancer MCF-7 and MDA-MB-231 cells. The results indicate that the anti-breast cancer activity of artemisinin trimers is generally superior to that of the corresponding dimers, and compared with the other three artemisinin trimers, compound **6a** exhibited the most potent IC_50_ value (MCF-7: IC_50_ = 0.09 ± 0.03 μM; MDA-MB-231: IC_50_ = 0.11 ± 0.06 μM). Therefore, in the following phenotypic experiments, we focused our continued research on the biological mechanism by which **6a** kills BC cells. 

**Figure 5:**
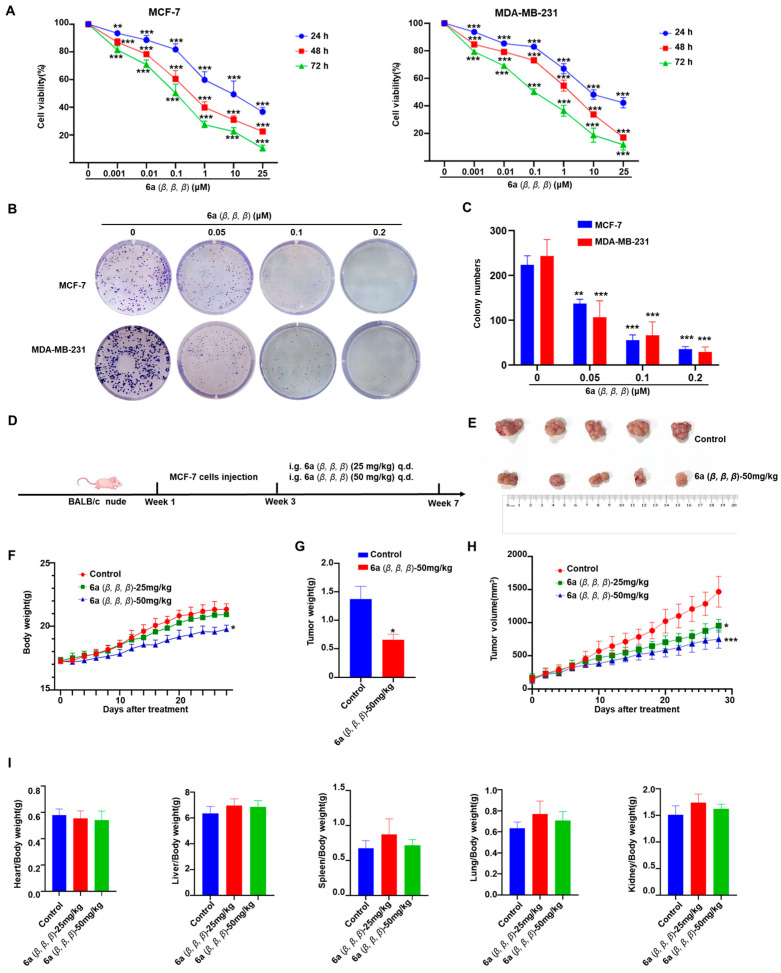
Compound **6a** inhibits BC growth in vitro and in vivo. (**A**) Dose- and time-dependent cytotoxicity of compound **6a** in MCF-7 and MDA-MB-231 cells was assessed by CCK-8 assay after 24, 48, and 72 h of treatment. Data are denoted as mean ± SD (*n* = 3). (**B**,**C**) Colony formation assays for MCF-7 and MDA-MB-231 cells treated with **6a** at the indicated concentrations. Colony numbers were quantified and are shown as mean ± SD (*n* = 3). (**D**) Schematic of the in vivo experimental design (*n* = 6 mice per group; i.g., intragastric administration). (**E**) Representative images of excised solid tumors from BALB/c nude mice throughout the treatment groups. (**F**) Body weight changes in mice during the study (mean ± SD, *n* = 6). (**G**) Final tumor weights from each group (mean ± SD, *n* = 6). (**H**) Tumor volume growth curves over the treatment period (mean ± SD, *n* = 6). (**I**) Organ indices calculated for different groups (mean ± SD, *n* = 6). * *p* < 0.05, ** *p* < 0.01, and *** *p* < 0.001 compared with the control group.

To investigate the anti-breast cancer efficacy of compound **6a** at the cellular level in vitro, we conducted plate cloning assays and cell growth curve experiments. Figure 5A demonstrates that the cytotoxic effect of compound **6a** on cell viability was contingent upon both its concentration and the duration of treatment. This inhibitory activity was corroborated by colony formation assays (Figure 5B,C), which simulate key aspects of in vivo tumor progression. In these assays, treatment with **6a** resulted in a concentration-dependent reduction in the clonogenic survival of MCF-7 and MDA-MB-231 cells. Results indicate that compound **6a** exhibits potent antitumor cell proliferation activity in vitro. 

To assess the in vivo anti-cancer viability of **6a**, MCF-7 cells were subcutaneously implanted into female BALB/C nude mice [30,31]. The treatment of MCF-7 tumors in these mice with different concentrations of **6a** (25 mg/kg, 50 mg/kg) (Figure 5D) resulted in significantly decreased tumor growth. Specifically, all treatment groups receiving **6a** demonstrated a clear inhibition of tumor growth (Figure 5E). Throughout the treatment period, mice administered 25 mg/kg exhibited relatively stable body weight gain compared to the control group. However, those receiving the higher dose of 50 mg/kg showed a slower rate of weight gain (Figure 5F). After euthanizing the nude mice, the tumors were weighed and photographed. Compared to controls, administration of **6a** at 50 mg/kg significantly decreased tumor size and weight (Figure 5G,H). Additionally, livers, lungs, hearts, kidneys, and spleens were harvested, with the organ coefficients calculated. No significant differences in organ coefficients were observed among the experimental groups (Figure 5I). The results indicate that **6a** exhibits promising in vivo anti-breast cancer efficacy.

### 2.5. The Effect of **6a** (β, β, β) on Cell Cycle Arrest

**Figure 6:**
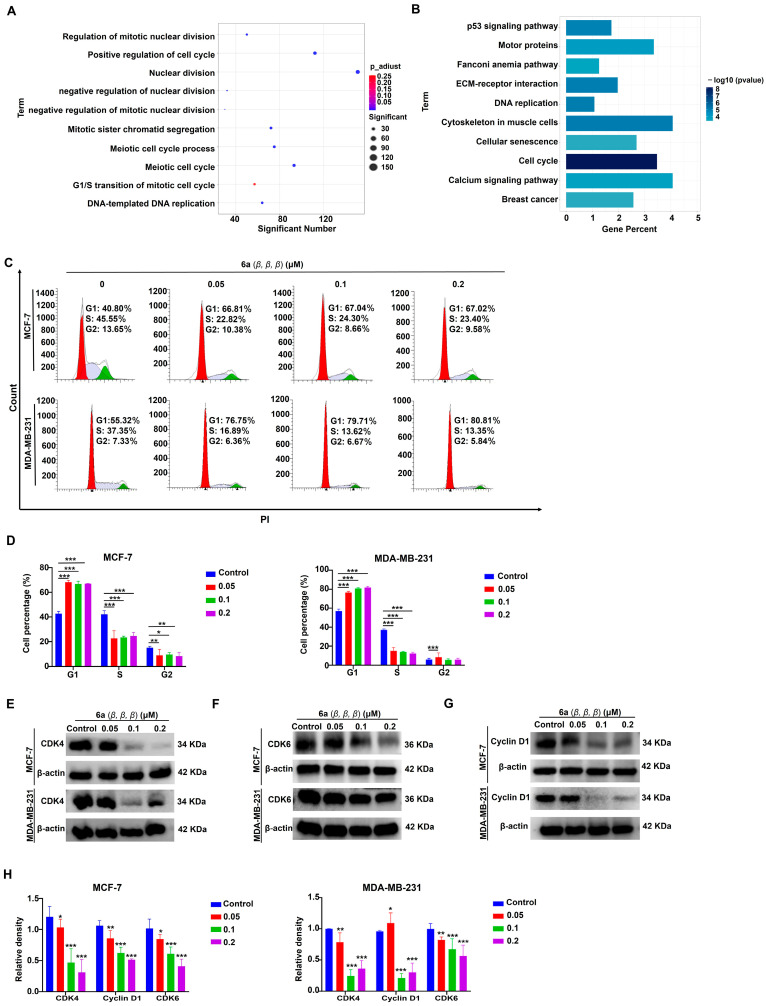
Effects of **6a** on cell cycle progression in breast cancer cell lines. (**A**,**B**) Results of KEGG and GO enrichment analyses for identified differentially expressed genes. (**C**,**D**) Cells were exposed to **6a** at specified doses for 24 h or left untreated. Subsequent cell cycle profiling was performed via flow cytometry following PI labeling. (**E**–**G**) Western blot analysis of CDK4 (**E**), CDK6 (**F**) and Cyclin-D1 (**G**) protein levels in MCF-7 and MDA-MB-231 cells treated with **6a** (*β*, *β*, *β*) for 48 h. β-actin served as the internal control. (**H**) Statistical analysis of (**E**–**G**). * *p* < 0.05, ** *p* < 0.01, and *** *p* < 0.001 compared with the control group.

In previous studies, we proved that **6a** (*β*, *β*, *β*) promotes apoptosis and ferroptosis in the BC cell line MCF-7 [29]. To further investigate the therapeutic targets and mechanisms of **6a** in breast cancer, we conducted a transcriptomic analysis using the MCF-7 BC cell line before and after treatment with **6a**. Figure 6A,B display the results of GO and KEGG enrichment analyses from the transcriptomics study. Our data reveal that the meiotic cell cycle signaling pathway exhibited the highest enrichment of differentially regulated genes. Cell cycle arrest is a primary mechanism underlying the cancer cell-killing activity of many clinical chemotherapies. Therefore, flow cytometry analysis was performed to determine whether **6a** induces cycle arrest in MCF-7 cells. To explore the cell cycle-arresting effects of **6a** on BC cells, MCF-7 cells and MDA-MB-231 cells were processed with discrepant concentrations of **6a** (0.05, 0.1, and 0.2 μM). Subsequently, the cell cycle distribution of MCF-7 cells and MDA-MB-231 cells was examined, following the treatment with **6a**. As shown in Figure 6C,D, **6a** induced a significant G0/G1 phase arrest in MCF-7 cells and MDA-MB-231 cells. Cyclin-dependent kinases 4 and 6 serve as key mediators for the transition into the S phase of the cell cycle, playing crucial roles in the initiation, growth, and survival of various tumors [32,33]. The expression of Cyclin D1 is tightly regulated by multiple signaling pathways. As a critical regulator of the G1-S cell cycle transition, Cyclin D1 is overexpressed in numerous tumors [34]. Our data indicate that **6a** significantly reduces the expressions of Cyclin D1, CDK4 and CDK6 (Figure 6E–G).

## 3. Materials and Method

### 3.1. General Chemical Procedures

Reagents and solvents were procured from Sinopharm Group Co., Ltd. (Shanghai, China), Shanghai Titan Scientific Co., Ltd. (Shanghai, China), Shanghai Haohong Scientific Co., Ltd. (Shanghai, China), Sigma-Aldrich Co., LLC (Darmstadt, Germany), and Saan Chemical Technology (Shanghai) Co., Ltd. (Shanghai, China) and were employed without in-depth treatment. The processes of reactions were tracked by TLC on 300–400 mesh silica gel (Shanghai Titan Scientific Co., Ltd., Shanghai, China) using UV light at 254 or 365 nm for detection. Initial intermediates were structurally assigned based on ^1^H NMR or LRMS data. Comprehensive characterization of all target compounds involved ^1^H NMR, ^13^C NMR, and HRMS analyses. NMR measurements were executed on Bruker AVANCE NEO spectrometers (Bruker Corporation, Karlsruhe, Germany) (^1^H at 600, 500, or 400 MHz; ^13^C at 150, 125, or 100 MHz). Spectral data are reported with standard multiplicity abbreviations (s, d, t, q, m) and coupling constants in Hz. HRMS analyses were conducted on an Agilent 6545 LC/Q-TOF instrument (Agilent Technologies, Inc., Santa Clara, CA, USA). Specific Rotation was measured on Rudolph Research Analytical AUTOPOL IV Automatic Polarimeter (Rudolph Research Analytical, LLC, Hackettstown, NJ, USA). The 3D conformational models of compounds were generated using Chem3D Ultra (version 21.0). Initial 2D structures were converted into 3D coordinates and subjected to preliminary geometry optimization. A systematic conformational search was then performed using the MMFF94 force field to identify the global minimum-energy conformers. Energy minimization was carried out using a conjugate gradient algorithm with a convergence criterion of 0.001 kcal/mol·Å and a maximum of 2000 iterations. The resulting optimized 3D structures were employed to correlate interatomic distances with the experimentally observed NOESY cross-peaks.

### 3.2. Synthesis of Compounds

The compounds **2**, **3**, **4a**, **5a**, **6a** used in this study were described by our group as part of the exploration of novel anti-tumor active compounds [27]. Synthesis of **4b**, **4c**, **5b**, **6b**, **6c** and **6d** was described as follows. For general experimental data, see the Supporting Information.

#### 3.2.1. Compound **4b** (β, α)

Under nitrogen, a solution of compound **3** (10.0 g, 11.6 mmol) in anhydrous DMF (100 mL) was cooled to 0 °C. Then, piperidine (11.4 mL, 116 mmol) was supplemented drop by drop to the stirred solution. The reaction was maintained for 2 h at 0 °C, with consumption of the starting material monitored by TLC (eluent: DCM/MeOH = 15:1). Upon completion, the mixture was diluted with saturated brine (50 mL), forming a precipitate. This precipitate was isolated by filtration. The remaining filtrate was rinsed with brine (20 mL), dehydrated over anhydrous Na_2_SO_4_, and filtered. After concentrating the combined organic extracts under decreased pressure, the crude product underwent purification via silica gel column chromatography to yield compound **4b** (β, α) (1.6 g, 22%) as a white solid. ^1^H NMR (600 MHz, CDCl_3_) δ: 5.40 (s, 1H), 5.33 (s, 1H), 4.81 (d, J = 3.4 Hz, 1H), 4.45 (d, J = 9.3 Hz, 1H), 4.11–4.08 (m, 1H), 4.00–3.96 (m, 1H), 3.59–3.56 (m, 1H), 3.52–3.49 (m, 1H), 2.96–2.92 (m, 1H), 2.87–2.83 (m, 1H), 2.80–2.73 (m, 2H), 2.69–2.60 (m, 1H), 2.43–2.34 (m, 3H), 2.05–1.97 (m, 5H), 1.90–1.85 (m, 2H), 1.79–1.73 (m, 3H), 1.71–1.63 (m, 2H), 1.56–1.45 (m, 4H), 1.43 (s, 6H), 1.38–1.29 (m, 2H), 1.28–1.21 (m, 2H), 0.96 (d, J = 6.7 Hz, 6H), 0.89 (dd, J = 14.4, 7.3 Hz, 6H). ^13^C NMR (126 MHz, CDCl3) δ 104.2, 104.0, 102.1, 100.2, 91.1, 87.8, 81.0, 80.3, 68.4, 67.8, 53.5, 52.6, 51.6, 49.2, 49.1, 45.3, 44.4, 37.4, 37.3, 36.4, 36.3, 34.6, 34.2, 32.6, 30.9, 26.1, 26.0, 24.7, 24.6, 24.6, 22.1, 20.3, 20.2, 13.0, 12.6. HR-MS (ESI): calcd for C_34_H_55_NO_10_H ([M + H]^+^): 638.3904, found: 638.3910. Specific Rotation: ${\left[\alpha \right]}_{D}^{28.7}=+67.9\;(c\;0.20,MeOH)$ (Supporting Information page S3).

#### 3.2.2. Compound **4c** (α, α)

Under nitrogen protection, a solution of compound **3** (10.0 g, 11.6 mmol) in anhydrous DMF (100 mL) was stirred and cooled to 0 °C. Piperidine (11.4 mL, 116 mmol) was then introduced via dropwise addition. The reaction lasted for 2 h at this temperature, with its course followed by TLC (eluent: DCM/MeOH, 15:1). After the starting material was completely consumed, the reaction mixture was quenched by pouring it into saturated brine (50 mL). The produced mixture was obtained with EtOAc. Subsequently, the combined organic phases were rinsed with brine (20 mL), dehydrated over anhydrous Na_2_SO_4_, and screened. The filtrate concentration under decreased pressure yielded a crude residue, which then underwent purification through silica gel column chromatography to give compound **4c** (*α*, *α*) as a white solid (340 mg, 5%). ^1^H NMR (600 MHz, CDCl_3_) *δ*: 5.31 (s, 2H), 4.44 (d, *J* = 9.2 Hz, 2H), 4.06–4.03 (m, 2H), 3.60–3.57 (m, 2H), 2.91–2.87 (m, 4H), 2.79–2.76 (m, 2H), 2.40–2.31 (m, 4H), 2.02–1.97 (m, 2H), 1.88–1.83 (m, 2H), 1.76–1.64 (m, 4H), 1.53–1.42 (m, 4H), 1.41 (s, 6H), 1.32–1.20 (m, 7H), 1.13–1.10 (m, 1H), 0.93 (d, *J* = 6.2 Hz, 6H), 0.87 (d, *J* = 7.1 Hz, 6H). ^13^C NMR (126 MHz, CDCl_3_) *δ* 104.5, 100.9, 91.3, 80.4, 65.2, 51.6, 48.2, 45.4, 37.4, 36.3, 34.2, 32.6, 26.1, 24.7, 22.1, 20.3, 12.7. HR-MS (ESI): calcd for C_34_H_55_NO_10_H ([M + H]^+^): 638.3904, found: 638.3911 (Supporting Information page S4).

#### 3.2.3. Compound **5b**

Within a dehydrated three-necked flask, a mixture of DHA (3.0 g, 10.6 mmol) and 2-bromoethanol (1.32 g, 10.6 mmol) was dissolved in anhydrous DCM (80 mL) under nitrogen. The solution was cooled to 0 °C before BF_3_·Et_2_O (744 mg, 5.28 mmol) was supplemented drop by drop. Agitation lasted for 2 h at 25 °C. The reaction progress was monitored via TLC (PE/EA = 5:1). Upon completion, the mixture was quenched by supplementing saturated aqueous NaHCO_3_ (30 mL). After that, the produced mixture was obtained three times with DCM (20 mL each). The combined organic phases were rinsed with brine (50 mL), dehydrated over anhydrous Na_2_SO_4_, and condensed under decreased pressure. Subsequent purification of the crude product via silica gel column chromatography yielded compound **5b** as a colorless oil (102 mg, 2%). ^1^H NMR (400 MHz, CDCl_3_) δ: 5.51 (s, 1H), 5.03 (d, J = 4.6 Hz, 1H), 4.19–4.12 (m, 1H), 3.91–3.85 (m, 1H), 3.58–3.51 (m, 2H), 2.37–2.29 (m, 1H), 2.07–2.02 (m, 1H), 1.95–1.77 (m, 2H), 1.63–1.57 (m, 3H), 1.46–1.40 (m, 5H), 1.30–1.28 (m, 1H), 1.24 (d, J = 7.3 Hz, 3H), 0.97 (d, J = 6.0 Hz, 3H), 0.92–0.85 (m, 2H). HR-MS (ESI): calcd for C_17_H_27_BrO_5_Na ([M + Na]^+^): 413.0940, found: 413.0939 (Supporting Information page S5).

#### 3.2.4. Compound **6b** (β, β, α)

Within a round-bottom flask, compound **4b** (100 mg, 0.16 mmol) and compound **5a** (73.6 mg, 0.19 mmol) were dissolved in anhydrous DMF (4 mL). To this solution, K_2_CO_3_ (65 mg, 0.47 mmol) was added under a nitrogen atmosphere. The resulting mixture was stirred at 40 °C for 48 h, with reaction progress monitored by TLC (PE/EA = 1:1). After completion was confirmed, the reaction was quenched by the addition of saturated aqueous NH_4_Cl. The aqueous mixture was extracted three times with ethyl acetate (30 mL each). The combined organic extracts were sequentially rinsed with brine, dehydrated over anhydrous Na_2_SO_4_, and condensed under decreased pressure. Finally, the crude product underwent purification through silica gel column chromatography, yielding compound **6b** (β, β, α) as a white solid (91 mg, 61%). ^1^H NMR (400 MHz, CDCl_3_) δ 5.38 (s, 2H), 5.30 (s, 1H), 4.75 (d, J = 3.1 Hz, 2H), 4.41 (d, J = 9.2 Hz, 1H), 4.00–3.94 (m, 1H), 3.89–3.83 (m, 2H), 3.49–3.41 (m, 2H), 2.79–2.72 (m, 5H), 2.61–2.57 (m, 2H), 2.38–2.30 (m, 4H), 2.02–1.99 (m, 4H), 1.87–1.84 (m, 4H), 1.77–1.60 (m, 8H), 1.53–1.47 (m, 3H), 1.41 (s, 11H), 1.33–1.18 (m, 9H), 0.93 (d, J = 6.2 Hz, 9H), 0.89–0.83 (m, 10H). ^13^C NMR (100 MHz, CDCl_3_) δ 104.2, 104.0, 102.2, 100.3, 91.2, 87.9, 81.1, 80.3, 67.8, 67.4, 54.7, 54.3, 52.6, 51.7, 45.4, 44.5, 37.4, 37.3, 36.5, 36.3, 34.7, 34.3, 32.6, 30.9, 26.2, 26.0, 24.7, 24.5, 22.2, 20.4, 20.3, 13.1, 12.7. HR-MS (ESI): calcd for C_51_H_81_NO_15_H ([M + H]^+^): 948.5684, found: 948.5738. Specific Rotation: ${\left[\alpha \right]}_{D}^{28.4}=+102.7\;(c\;0.20,MeOH)$ (Supporting Information pages S5 and S6).

#### 3.2.5. Compound **6c** (β, α, α)

Within a round-bottom flask, a solution of compound **4c** (100 mg, 0.16 mmol) and compound **5a** (73.6 mg, 0.19 mmol) in anhydrous DMF (4 mL) was prepared under a nitrogen atmosphere. Next, potassium carbonate (K_2_CO_3_, 65 mg, 0.47 mmol) was introduced into the mixture. The resulting suspension was continuously agitated for 2 days at 40 °C, with the reaction course followed by thin-layer chromatography (TLC) using a petroleum ether/ethyl acetate (1:1) solvent system. After the starting material was completely consumed, the reaction was terminated by adding saturated aqueous ammonium chloride (NH_4_Cl). The quenched mixture underwent extraction with ethyl acetate (three portions of 30 mL each). The organic phases were integrated together, rinsed with brine, dehydrated over anhydrous sodium sulfate (Na_2_SO_4_), and condensed using a rotary evaporator. Subsequently, the crude material obtained was purified via column chromatography on silica gel to isolate the product, yielding compound **6c** (β, α, α) as a white solid (90 mg, 61%). ^1^H NMR (400 MHz, CDCl_3_) δ 5.37 (s, 1H), 5.30 (s, 2H), 4.74 (d, J = 3.2 Hz, 1H), 4.41 (d, J = 9.2 Hz, 2H), 3.98–3.93 (m, 2H), 3.88–3.82 (m, 1H), 3.53–3.42 (m, 3H), 2.84–2.72 (m, 6H), 2.61–2.54 (m, 1H), 2.38–2.30 (m, 5H), 2.08–1.95 (m, 5H), 1.90–1.80 (m, 3H), 1.75–1.60 (m, 7H), 1.55–1.46 (m, 4H), 1.40 (s, 10H), 1.32–1.19 (m, 9H), 0.93 (d, J = 6.0 Hz, 9H), 0.88–0.83 (m, 10H). ^13^C NMR (100 MHz, CDCl_3_) δ104.3, 104.1, 102.3, 100.3, 91.2, 88.0, 81.2, 80.4, 67.8, 67.4, 54.9, 54.4, 52.7, 51.7, 45.4, 44.6, 37.5, 37.4, 36.6, 36.4, 34.7, 34.3, 32.7, 31.0, 26.3, 26.1, 24.8, 24.8, 24.5, 22.3, 20.5, 20.4, 13.2, 12.8. HR-MS (ESI): calcd for C_51_H_81_NO_15_H ([M + H]^+^): 948.5684, found: 948.5732. Specific Rotation: ${\left[\alpha \right]}_{D}^{28.7}=+51.5\;(c\;0.20,MeOH)$ (Supporting Information pages S6 and S7).

#### 3.2.6. Compound **6d** (α, α, α)

In a round-bottom flask under nitrogen, compounds **4c** (166 mg, 0.26 mmol) and **5b** (102 mg, 0.26 mmol) were digested within anhydrous DMF (4 mL). To this homogeneous solution, finely powdered potassium carbonate (K_2_CO_3_, 108 mg, 0.78 mmol) was incorporated in one portion. The resulting heterogeneous mixture was constantly agitated for 96 h at 40 °C. The progression of the reaction was tracked by thin-layer chromatography (mobile phase: petroleum ether/ethyl acetate, 1:1). After TLC analysis validated the complete disappearance of starting materials, the reaction was inhibited by carefully adding the saturated aqueous ammonium chloride. The resulting biphasic mixture was obtained thrice with ethyl acetate (3 × 30 mL). The pooled organic extracts were rinsed once with brine, dehydrated over anhydrous sodium sulfate, and the solvent was eliminated under decreased pressure. The crude residue thus obtained was subjected to purification by flash column chromatography on silica gel, which yielded compound **6d** (α, α, α) as a white solid (78 mg, 52%). ^1^H NMR (400 MHz, CDCl_3_) δ 5.44 (s, 1H), 5.32 (s, 2H), 4.94 (d, J = 4.2 Hz, 1H), 4.44 (d, J = 8.9 Hz, 2H), 4.04–3.88 (m, 3H), 3.61–3.49 (m, 3H), 2.88–2.77 (m, 5H), 2.40–2.29 (m, 5H), 2.01 (dt, J = 14.5, 3.7 Hz, 3H), 1.92 -1.84 (m, 3H), 1.77–1.66 (m, 7H), 1.62–1.59 (m, 2H), 1.56–1.46 (m, 6H), 1.42–1.41 (m, 10H), 1.33–1.24 (m, 9H), 1.18 (d, J = 7.3 Hz, 3H), 0.96–0.93 (m, 10H), 0.87 (d, J = 7.1 Hz, 6H). ^13^C NMR (100 MHz, CDCl_3_) δ 104.3, 103.2, 100.4, 91.3, 89.1, 81.7, 80.5, 67.8, 67.5, 54.7, 54.6, 52.1, 51.8, 46.6, 45.5, 37.5, 37.4, 36.7, 36.5, 34.6, 34.4, 32.8, 31.7, 26.2, 26.1, 24.9, 22.3, 20.4, 20.2, 19.7, 12.9. HR-MS (ESI): calcd for C_51_H_81_NO_15_H ([M + H]^+^): 948.5684, found: 948.5740 (Supporting Information pages S7 and S8).

### 3.3. Biological Methods

#### 3.3.1. Cell Viability Assay

To assess the cytotoxic effects of compound **6a**, an assay was employed using a commercial CCK-8 kit (Beyotime, Shanghai, China). MCF-7 and MDA-MB-231 (Cell Bank of the Chinese Academy of Sciences, Shanghai, China). cells were cultivated in 96-well plates at a density of 2 × 10^3^ cells per well and cultured overnight to ensure adhesion. Cells were then exposed to a concentration gradient of **6a** (0.001–25 μM) for durations of 24, 48, and 72 h. At each time point, 10 μL of CCK-8 reagent was introduced into each well, followed by a 1 h incubation at 37 °C. Absorbance readings were subsequently taken at a wavelength of 450 nm with a Tecan Spark multimode microplate reader.

#### 3.3.2. Colony Formation Assay

To evaluate the long-term proliferative capacity of breast cancer cells, a standard colony formation assay was conducted. MCF-7 and MDA-MB-231 cells were plated within 6-well plates at approximately 300 cells per well using conditioned medium and allowed to attach. Following a 24 h exposure to compound **6a** at doses of 0, 0.05, 0.1, and 0.2 μM, the medium was replenished with fresh complete medium. Afterward, cells were cultured for another 24 h in standard culture conditions (37 °C, 5% CO_2_). Thereafter, the monolayers were rinsed with PBS, immobilized using 4% paraformaldehyde, and stained with a 0.5% crystal violet solution (Yeasen, Shanghai, China). Lastly, colonies were enumerated manually.

#### 3.3.3. Animal Experiments

All animal procedures were reviewed and received approvals from the Animal Ethics Committee of Shanghai University of Traditional Chinese Medicine (Approval Number: PZSHUTCM2406130001).

Four-week-old female BALB/c nude mice, from Shanghai Jihui Laboratory Animal Breeding Co., Ltd. (Shanghai, China), underwent a one-week acclimatization. Subsequently, MCF-7 xenograft models were established by subcutaneous injection of 5 × 10^6^ cells suspended in a 1:1 Matrigel mixture. Tumor development was supported by weekly estradiol benzoate (Proteintech, Wuhan, China) injections (100 µg/mouse). Two weeks post-inoculation, when tumor volumes reached 100–200 mm^3^, the mice were allocated stochastically into three groups (*n* = 6). Treatments included: oral gavage of 0.5% CMC-Na (vehicle control), oral administration of compound **6a** at 25 or 50 mg/kg/day. Animal body weights and tumor dimensions (volume calculated as ½ × length × width^2^) were recorded every other day throughout the four-week treatment period. Upon termination, mice were euthanized, and major organs were harvested for organ coefficient calculation.

#### 3.3.4. Transcriptome Sequencing and Analysis

Cell Culture and Treatment: MCF-7 and MDA-MB-231 human breast cancer cell lines were cultured in DMEM (Dulbecco’s Modified Eagle Medium). The culture medium was supplemented with 10% (*v*/*v*) fetal bovine serum (FBS) and 1% penicillin-streptomycin antibiotic solution. Cells were preserved within a humid incubator under 5% CO_2_ at 37 °C. For the experiment, cells were seeded at an appropriate density and treated with either 0.1 μM compound **6a** or 0.1% DMSO (vehicle control) for 48 h upon reaching approximately 70–80% confluence.

RNA Extraction and Quality Control: Total RNA was isolated from the harvested cells via TRIzol reagent (Invitrogen, Carlsbad, CA, USA). The concentration and purity of the RNA were appraised via NanoDrop spectrophotometer, and RNA integrity was evaluated using an Agilent 2100 Bioanalyzer (Agilent Technologies, Santa Clara, CA, USA). All RNA samples used for library preparation had an A260/A280 ratio between 1.8 and 2.0 and an RNA Integrity Number (RIN) ≥ 7.0.

Library Preparation and Sequencing: Sequencing libraries were constructed from 1 μg of total RNA per sample using the NEBNext^®^ Ultra™ II Directional RNA Library Prep Kit for Illumina (New England Biolabs, Ipswich, MA, USA) according to the manufacturer’s recommendations. The final library quality was assessed using the Agilent 2100 Bioanalyzer. Subsequently, the qualified libraries were sequenced on an Illumina NovaSeq 6000 platform (Illumina, San Diego, CA, USA) to produce 150 bp paired-end reads.

Bioinformatic Analysis: Raw sequencing data (raw reads) were processed through quality control checks using Fast QC. Following the removal of adapter sequences and low-quality reads via Trimmomatic, the resulting clean reads were mapped to the GRCh38 human reference genome using HISAT2. Differential gene expression analysis between the control and compound **6a**-treated groups was performed using the DESeq2 R package (version 1.34.0). Genes with an adjusted *p*-value (FDR) < 0.05 and |log_2_(FoldChange)| > 1 were considered significantly differentially expressed.

#### 3.3.5. Cell Cycle Analysis

MCF-7 and MDA-MB-231 cells were seeded in 6 cm dishes and treated with the indicated concentrations (0, 0.05, 0.1, and 0.2 μM) of compound **6a** for 24 h. Following trypsinization and centrifugation, the harvested cells were fixed and permeabilized in ice-cold 70% ethanol for 24 h at 4 °C. The fixed cells were then thoroughly rinsed with PBS and subsequently stained for 30 min in darkness at 37 °C using a PBS solution containing RNase and propidium iodide (PI). Cell cycle distribution was analyzed by flow cytometry. The entire experiment was performed in triplicate across three independent trials. The cell cycle detection kit was procured from Beyotime (Shanghai, China).

#### 3.3.6. Western Blot

For Western blot analysis, cells treated with compound **6a** were lysed on ice using RIPA Lysis Buffer (Yeasen, Shanghai, China) added with protease and phosphatase inhibitor cocktails (Yeasen). Protein concentrations in the resultant lysates were determined with a BCA Protein Assay Kit (Beyotime, Shanghai, China). Equal protein quantities were resolved via sodium dodecyl sulfate-polyacrylamide gel electrophoresis (SDS-PAGE) and electrophoretically delivered to PVDF membranes. These membranes were first cultivated for 1 h at room temperature in a blocking buffer consisting of 5% non-fat dry milk prepared in TBST (Tris-buffered saline with 0.1% Tween-20). Next, they were nurtured all night at 4 °C with the indicated primary antibodies. Following three 10 min TBST washes, membranes were then cultivated for 2 h at ambient temperature with horseradish peroxidase (HRP)-conjugated secondary antibodies. After a final wash cycle with TBST, immunoreactive bands were developed using an enhanced chemiluminescence (ECL-Plus) substrate and captured on imaging equipment.

#### 3.3.7. Statistical Analysis

The data are reported as mean ± standard deviation (SD). Statistical significance was evaluated with GraphPad Prism 9.0.0. Inter-group differences were assessed using an unpaired *t*-test for two-group comparisons or one-way ANOVA for multi-group comparisons, followed by post hoc tests where appropriate. Tumor growth kinetics were analyzed by two-way repeated-measures ANOVA. Image-based quantifications (e.g., from blot or colony assays) were executed using ImageJ software version 1.53k (National Institutes of Health, Bethesda, MD, USA). 

## 4. Conclusions

In this study, three distinct configurational isomers of artemisinin trimer, **6b** (*β*, *β*, *α*), **6c** (*β*, *α*, *α*), and **6d** (*α*, *α*, *α*), were synthesized and structurally characterized for the first time. The confirmation of molecular structures relied on comprehensive spectroscopic data, including ^1^H NMR, ^13^C NMR, ^1^H-^1^H COSY, ^1^H-^1^H NOESY, HMBC, and HMQC. The antitumor activities of four trimer configurations were systematically evaluated in BC cell lines MCF-7 and MDA-MB-231. Among these compounds, **6a** (*β*, *β*, *β*) shows good antitumor activity against both cell lines, so we chose it for in vivo antitumor activity analysis. The results indicate that 6a not only has good in vitro antitumor activity, but also effectively inhibits tumor growth in xenografted nude mice. Further mechanistic studies indicate that **6a** (*β*, *β*, *β*) exerts antitumor effects via inhibiting the production of the CyclinD1-CDK4/6 complex in MCF-7 BC cells and arresting the MCF-7 cell cycle in the G0/G1 stage.

Overall, this work establishes stereochemistry as a critical determinant of the antitumor activity of artemisinin trimers. The observed stereoselective differences among configurational variants provide mechanistic insight into structure–activity relationships within this class of compounds and offer a rational basis for the development of artemisinin-based antitumor agents. Future studies will focus on elucidating the molecular interactions underlying this stereoselectivity and further assessing the therapeutic potential of the (*β*, *β*, *β*) trimer as a lead compound for targeted cancer therapy.

## Figures and Tables

**Table 1:** NMR spectral data for compounds **6a** (*β*, *β*, *β*), **6b** (*β*, *β*, *α*), **6c** (*β*, *α*, *α*) and **6d** (*α*, *α*, *α*).

Position	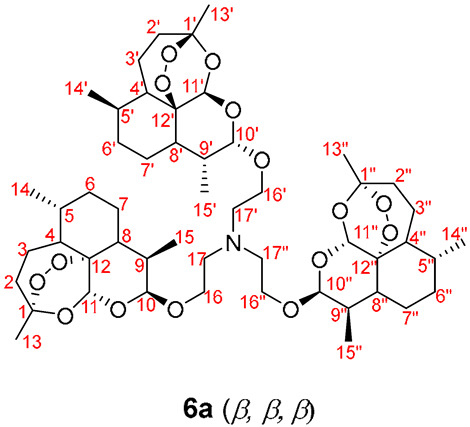		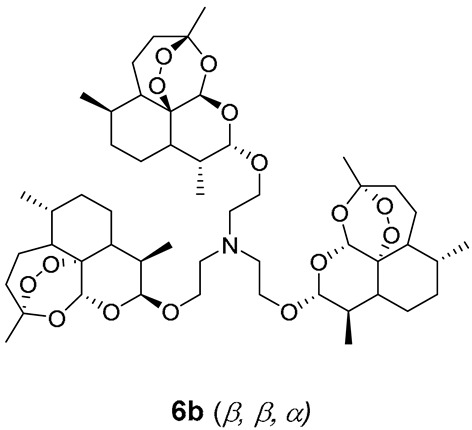		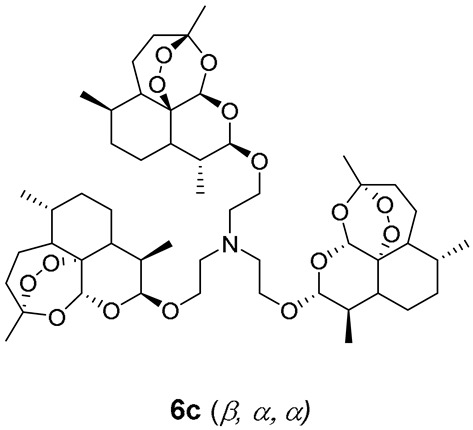		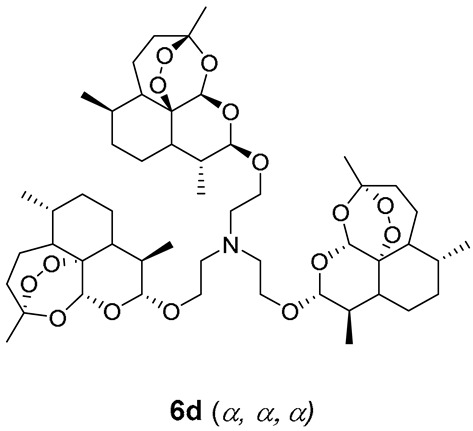	
	*δ*H Mult (*J* in Hz)	*δ*C	*δ*H Mult (*J* in Hz)	*δ*C	*δ*H Mult (*J* in Hz)	*δ*C	*δ*H Mult (*J* in Hz)	*δ*C
1/1′/1″	/	104.5 104.3	/	104.2 104.0	/	104.3 104.1	/	104.3
2/2′/2″	2.36 td, (14.1, 3.9) 2.04–2.00, m	36.6 36.4	2.38–2.30, m 2.02–1.99, m	36.5 36.3	2.38–2.30, m 2.08–1.95, m	36.6 36.4	2.40–2.29, m 2.01 dt, (14.5, 3.7)	36.7 36.5
3/3′/3″	1.89–1.85, m 1.52–1.47, m	24.8 24.8 24.5	1.87–1.84, m 1.53–1.47, m	24.7 24.5	1.90–1.80, m 1.55–1.46, m	24.8 24.8	1.92–1.84, m 1.56–1.46, m	24.9
4/4′/4″	1.25–1.22, m	52.7 52.4	1.33–1.18, m	52.6 51.7	1.32–1.19, m	52.7 51.7	1.33–1.24, m	52.1 51.8
5/5′/5″	1.35–1.30, m	37.5 37.4	1.33–1.18, m	37.4 37.3	1.32–1.19, m	37.5 37.4	1.33–1.24, m	37.5 37.4
6/6′/6″	1.65–1.61, m	34.7 34.3	1.77–1.60, m	34.7 34.3	1.75–1.60, m	34.7 34.3	1.77–1.66, m	34.6 34.4
7/7′/7″	1.80–1.70, m	24.8 24.8 24.5	1.87–1.84, m	22.2	1.90–1.80, m	22.3	1.92–1.84, m	22.3
8/8′/8″	1,45–1.43, m	44.5	1.53–1.47, m	45.4 44.5	1.55–1.46, m	45.4 44.6	1.56–1.46 m	46.6 45.5
9/9′/9″	2.62–2.59, m	30.9	2.61–2.57, m	32.6 30.9	2.61–2.54, m	32.7 31.0	2.40–2.29, m	32.8 31.7
10/10′/10″	4.76, d, (3.3)	102.6 100.6	4.75, d, (3.1) 4.41, d, (9.2)	102.2 100.3	4.74, d, (3.2) 4.41, d, (9.2)	102.3 100.3	4.94, d, (4.2) 4.44, d, (8.9)	103.2 100.4
11/11′/11″	5.39, s	88.1	5.38, s 5.30, s	87.9 91.2	5.37, s 5.30, s	88.0 91.2	5.44, s 5.32, s	91.3 89.1
12/12′/12″	/	81.2 80.5	/	81.1 80.3	/	81.2 80.4	/	81.7 80.5
13/13′/13″	1.42, s	26.3 26.1	1.41, s	26.2 26.0	1.40, s	26.3 26.1	1.42, d, (6.4)	26.2 26.1
14/14′/14″	0.95, d, (6.3)	20.5 20.4	0.93, d, (6.2)	20.4 20.3	0.93, d, (6.0)	20.5 20.4	0.96–0.93, m	20.4 20.2
15/15′/15″	0.89, d, (7.3)	12.7 13.2	0.89–0.83, m	13.1 12.7	0.88–0.83, m	13.2 12.8	0.87, d, (7.1) 1.18, d, (7.3)	12.9 19.7
16/16′/16″	3.92–3.86, m 3.45–3.42, m	66.6 65.8	3.89–3.83, m 3.49–3.41, m	67.8 67.4	3.98–3.93, m 3.88–3.82, m 3.53–3.42, m	67.8 67.4	4.04–3.88, m 3.61–3.49, m	67.8 67.5
17/17′/17″	2.82–2.69, m	51.7	2.79–2.72, m	54.7 54.3	2.84–2.72, m	54.9 54.4	2.88–2.77, m	54.7 54.6

Spectra were acquired on a commercially available instrument recording in CDCl_3_ at 400, 500, 600 MHz for ^1^H NMR and 100, 125, 150 MHz for ^13^C NMR.

**Table 2:** The impact of compounds in MCF-7 and MDA-MB-231 cells.

Compound	IC_50_/(μM)	
	MCF-7	MDA-MB-231
**4a** (*β*, *β)*	3.62 ± 0.05	6.24 ± 1.06
**4b** (*β*, *α*)	0.33 ± 0.04	5.25 ± 2.79
**4c** (*α*, *α*)	2.70 ± 0.06	>10
**6b** (*β*, *β*, *α*)	0.03 ± 0.005	>10
**6c** (*β*, *α*, *α*)	0.05 ± 0.002	3.89 ± 0.02
**6d** (*α*, *α*, *α*)	0.06 ± 0.01	2.85 ± 0.24
**6a** (*β*, *β*, *β*)	0.09 ± 0.03	0.11 ± 0.06
Cisplatin	2.41 ± 0.27	8.57 ± 1.83
Gemcitabine	4.33 ± 0.36	0.73 ± 0.07

## Data Availability

The data in favor of our research findings are available in the paper’s Supplementary Information Files.

## Supplementary Materials

The following supporting information can be downloaded at: https://www.mdpi.com/article/10.3390/molecules31081228/s1, ^1^H and ^13^C NMR spectral data of compound **4b**; ^1^H and ^13^C NMR spectral data of compound **4c**; ^1^H NMR spectral data of compound **5b**; ^1^H and ^13^C NMR spectral data of compound **6b**; ^1^H and ^13^C NMR spectral data of compound **6c**; ^1^H and ^13^C NMR spectral data of compound **6d**; ^1^H-^1^H COSY, HMQC, HMBC and ^1^H-^1^H NOESY of compound **6b**; ^1^H-^1^H COSY, HMQC, HMBC and ^1^H-^1^H NOESY of compound **6c**; ^1^H-^1^H COSY, HMQC, HMBC and ^1^H-^1^H NOESY of compound **6d**.
