# Supplementary material for: Synthesis and Biological Evaluation of Isomeric Artemisinin Trimers as Novel Antitumor Agents
Source: Molecules. 2026 Apr 8;31(8):1228. doi: 10.3390/molecules31081228 (PMC13118418; doi:10.3390/molecules31081228)
Supplement: Supplementary file 1 [file molecules-31-01228-s001.zip › molecules-4193358-supplementary.pdf]

Supporting Information

Synthesis and Biological Evaluation of Isomeric Artemisinin Trimers as Novel Antitumor Agents

Zejin Zhang [1,†], Along Li [2,3,†], Bingying Jiang [4,†], Typhaine Bejoma [1],

Yongxi Zhao [1], Fujiang Guo [4], Yajuan Li [2,*], Huiyu Li [5,*] and Qingjie Zhao [1,*]

Table of Contents Page

1. ^{1}H NMR spectral data of compound **4b** S3
2. ^{1}H NMR spectral data of compound **4c** S3
3. ^{1}H NMR spectral data of compound **5b** S4
4. ^{1}H and ^{13}C NMR spectral data of compound **6b** S4-S5
5. ^{1}H and ^{13}C NMR spectral data of compound **6c** S5-S6
6. ^{1}H and ^{13}C NMR spectral data of compound **6d** S6-S7
7. ^{1}H-^{1}H COSY, HMQC, HMBC and ^{1}H-^{1}H NOESY of compound **6b** S7-S9
8. ^{1}H-^{1}H COSY, HMQC, HMBC and ^{1}H-^{1}H NOESY of compound **6c** S9-S11
9. ^{1}H-^{1}H COSY, HMQC, HMBC and ^{1}H-^{1}H NOESY of compound **6d** S11-S13

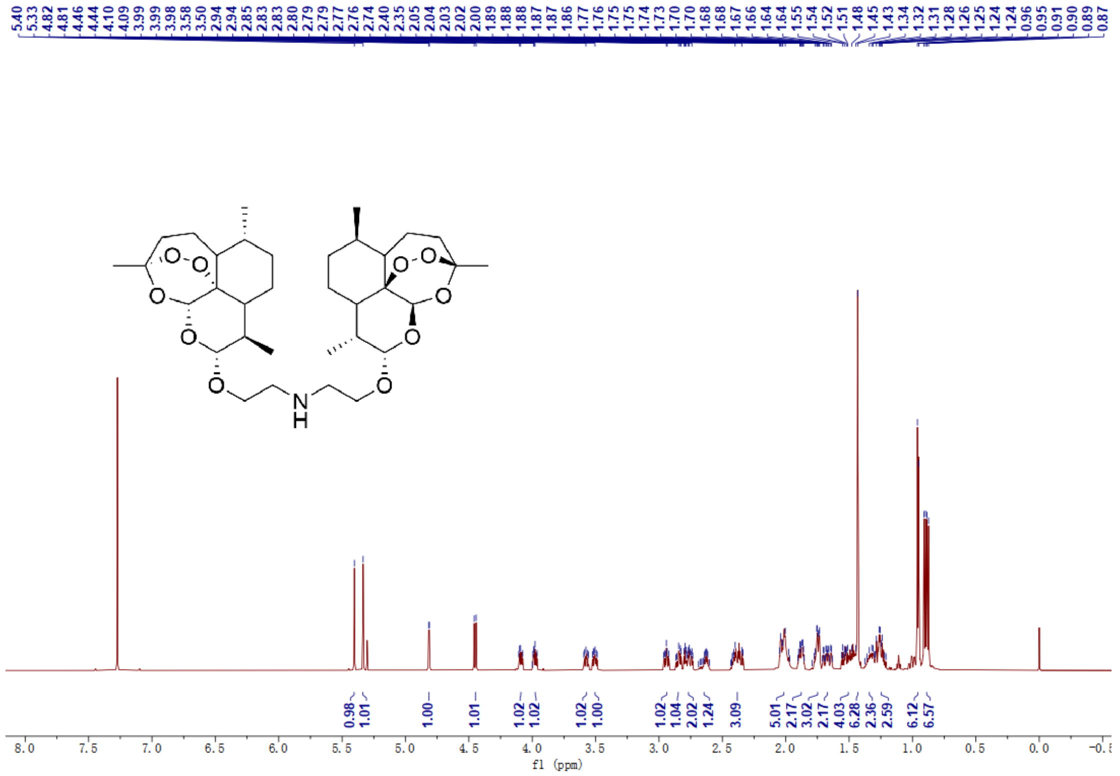

^{1}H NMR spectral data of compound **4b** (*β*, *α*)

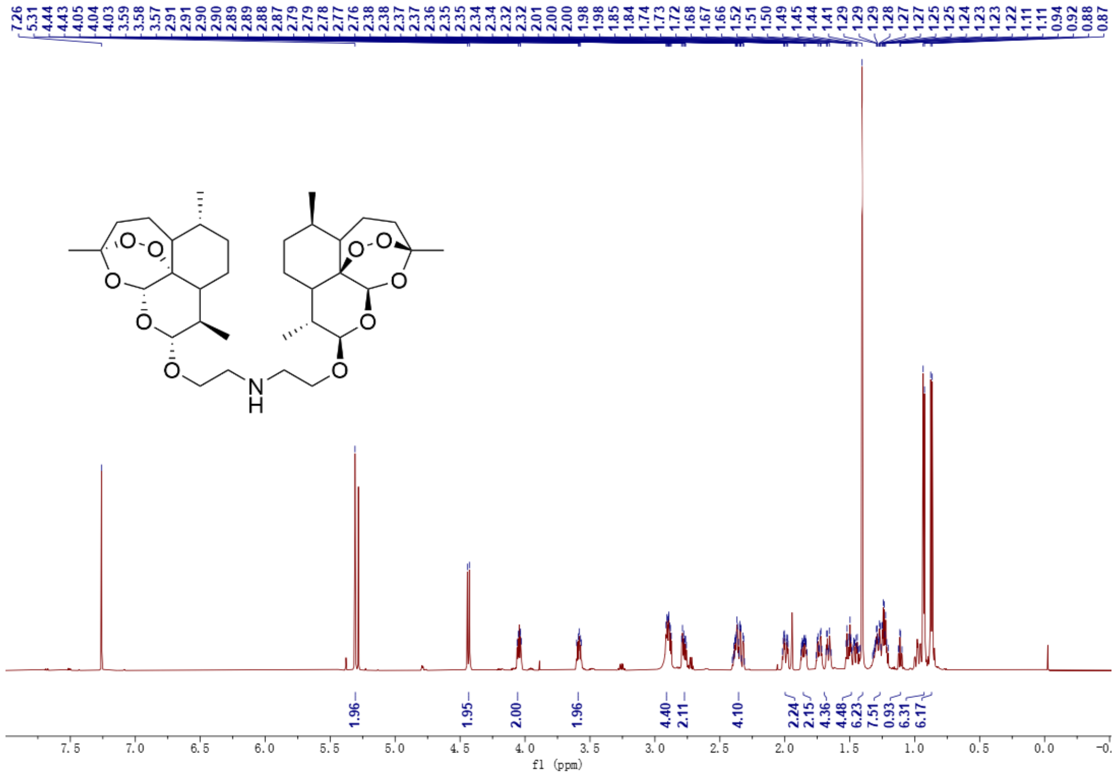

^{1}H NMR spectral data of compound **4c** (*α*, *α*)

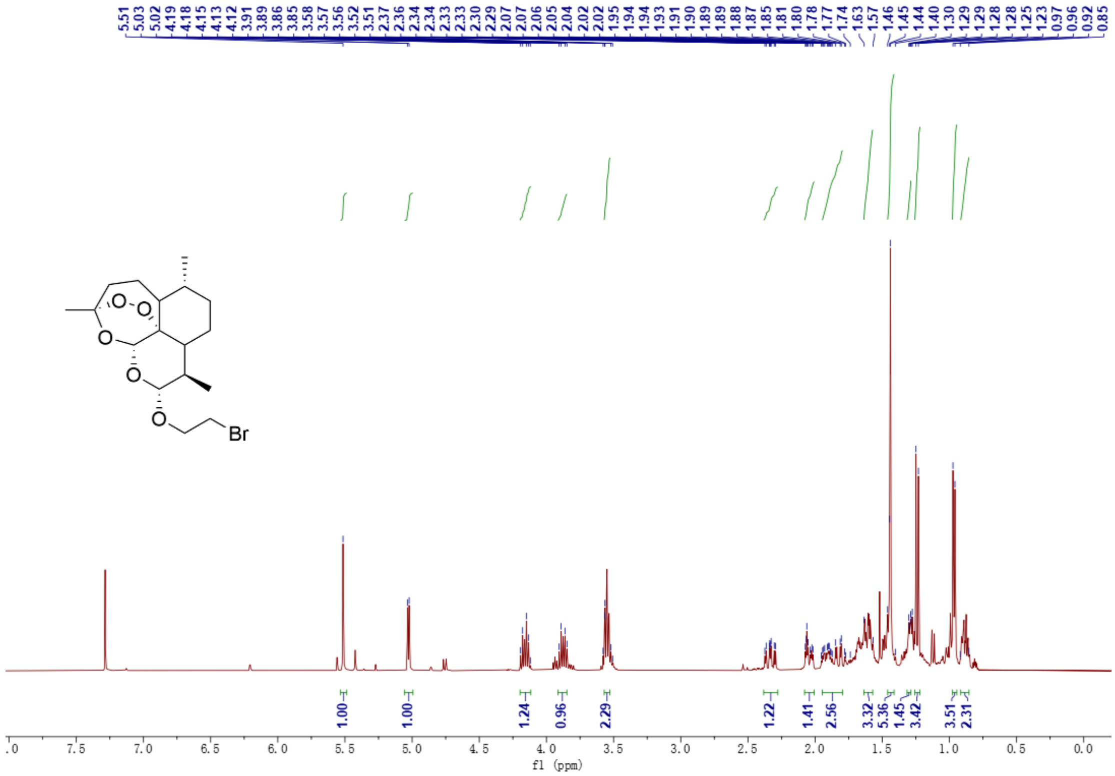

^{1}H NMR spectral data of compound **5b**

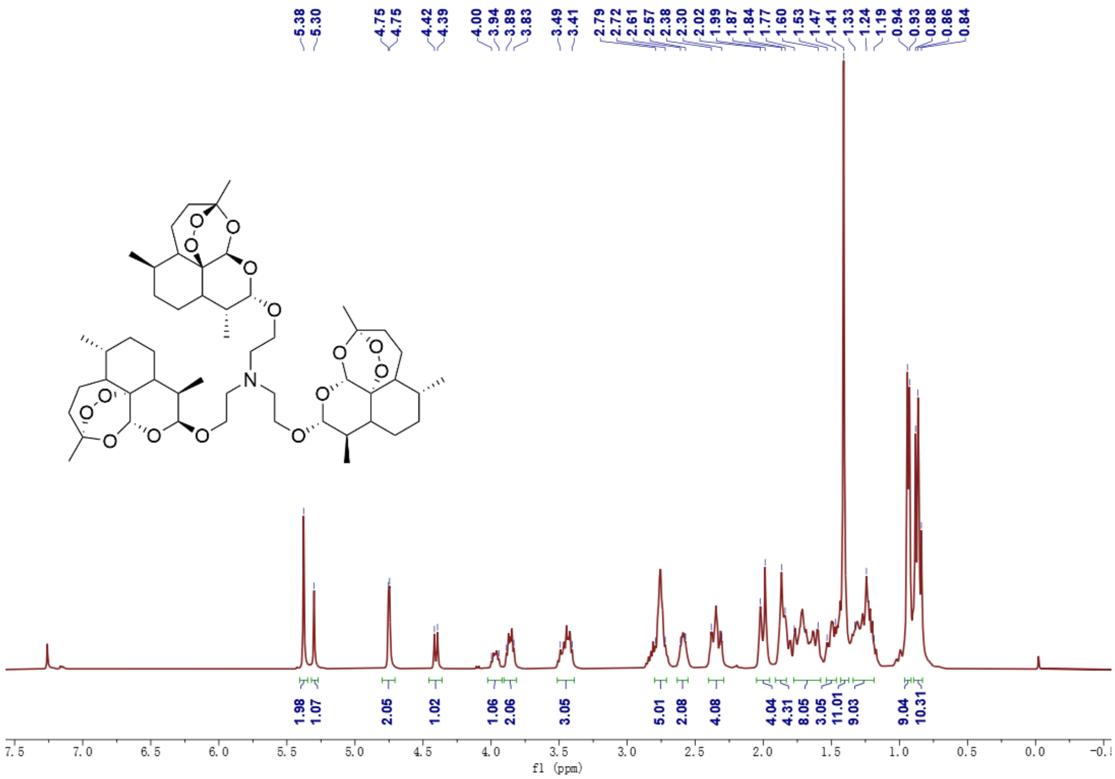

^{1}H NMR spectral data of compound **6b** (*β*, *β*, *α*)

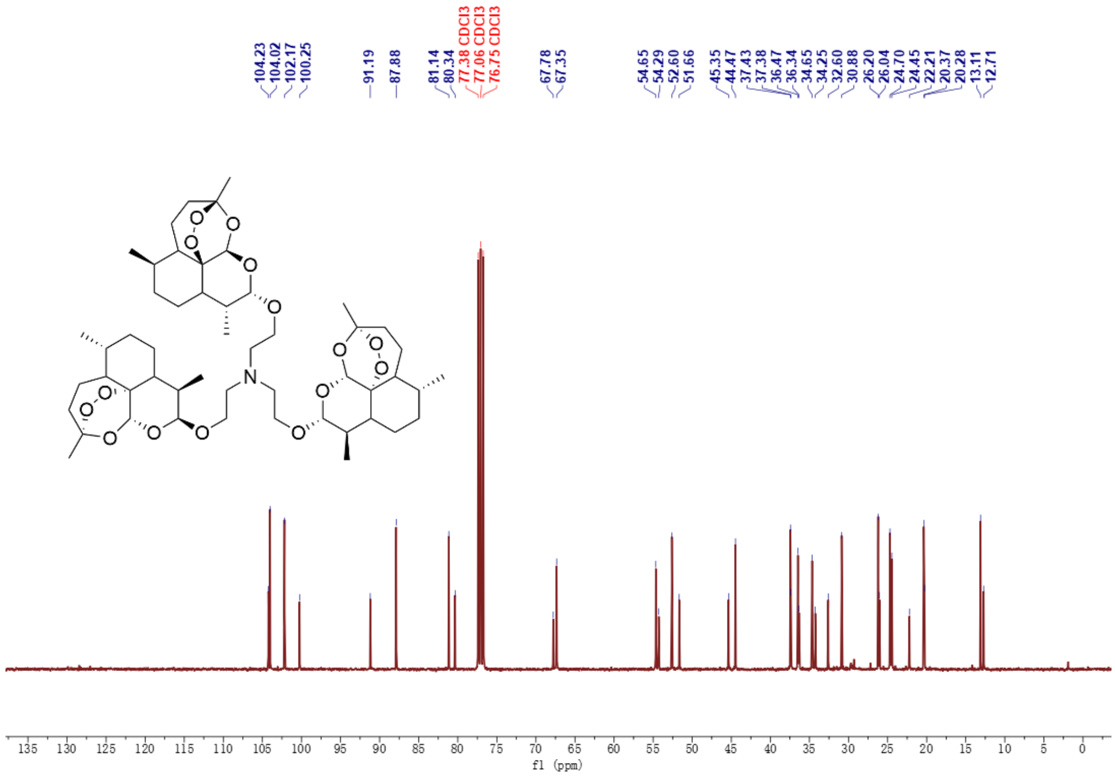

^{13}C NMR spectral data of compound **6b** (*β, β, α*)

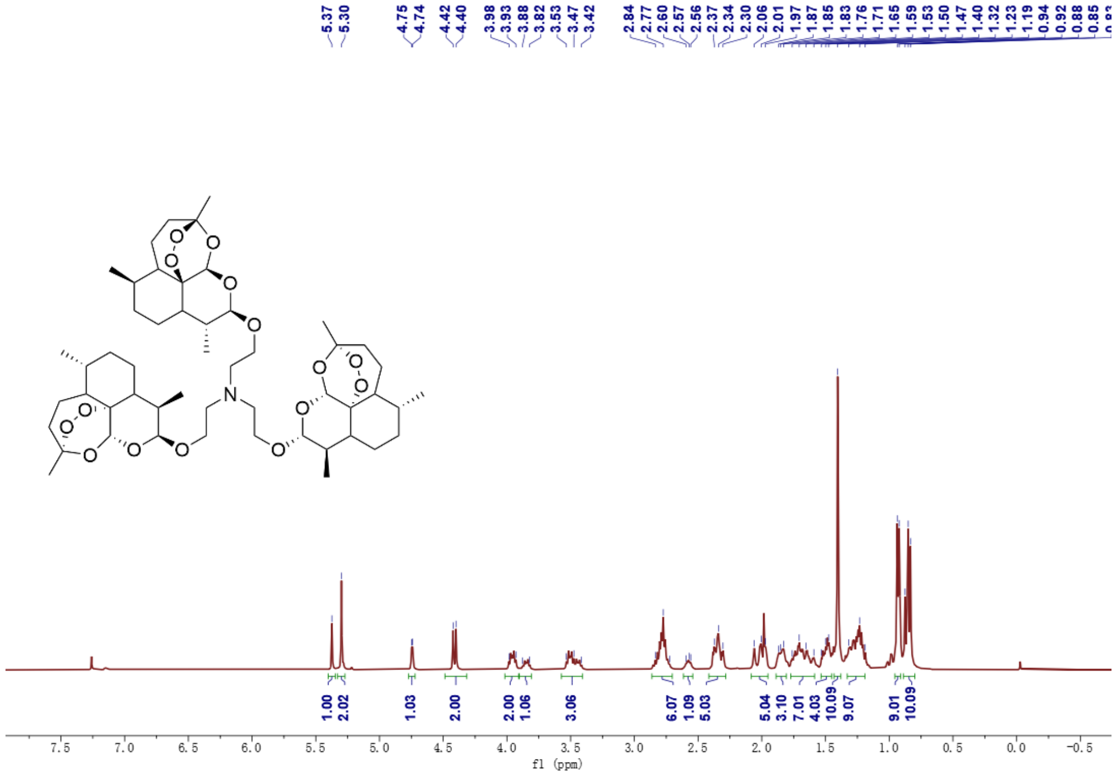

^{1}H NMR spectral data of compound **6c** (*β, α, α*)

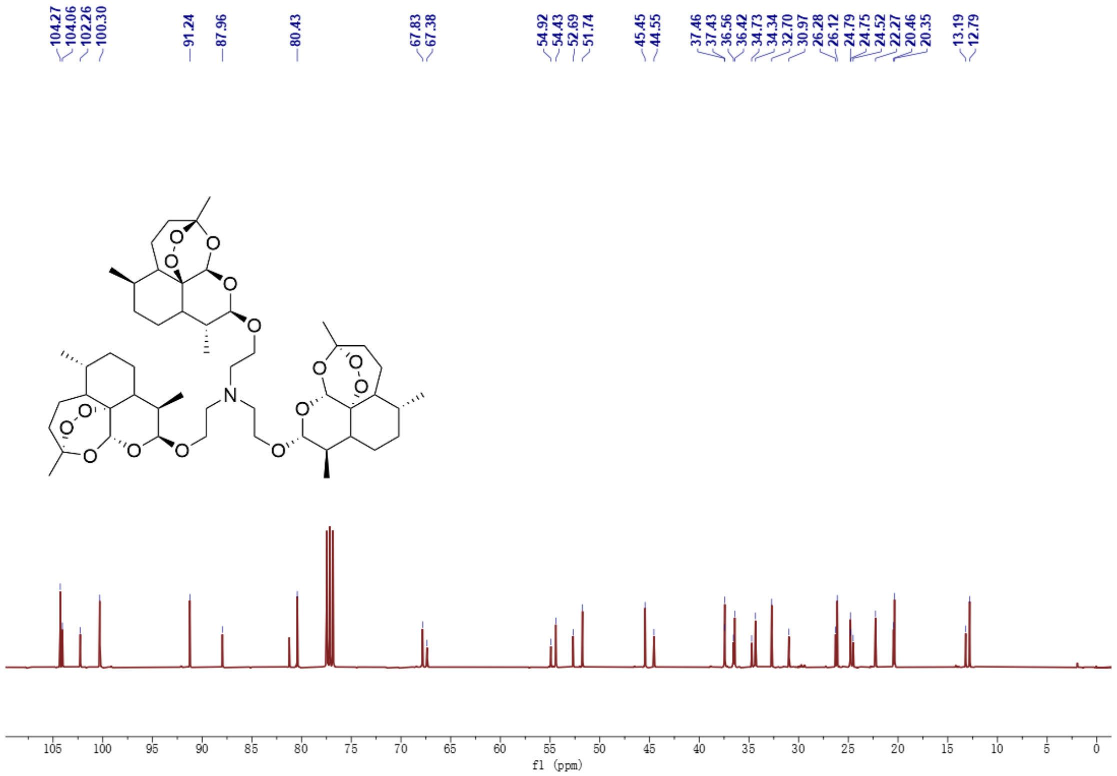

^{13}C NMR spectral data of compound **6c** (*β, α, α*)

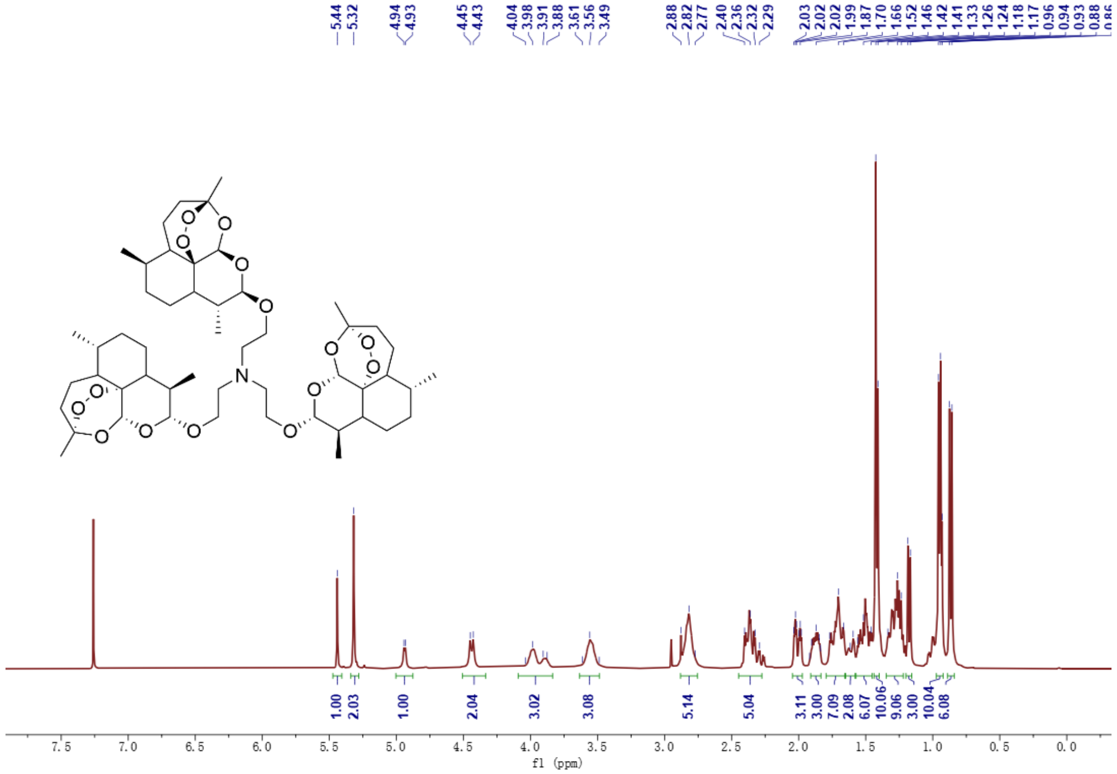

^{1}H NMR spectral data of compound **6d** (*α, α, α*)

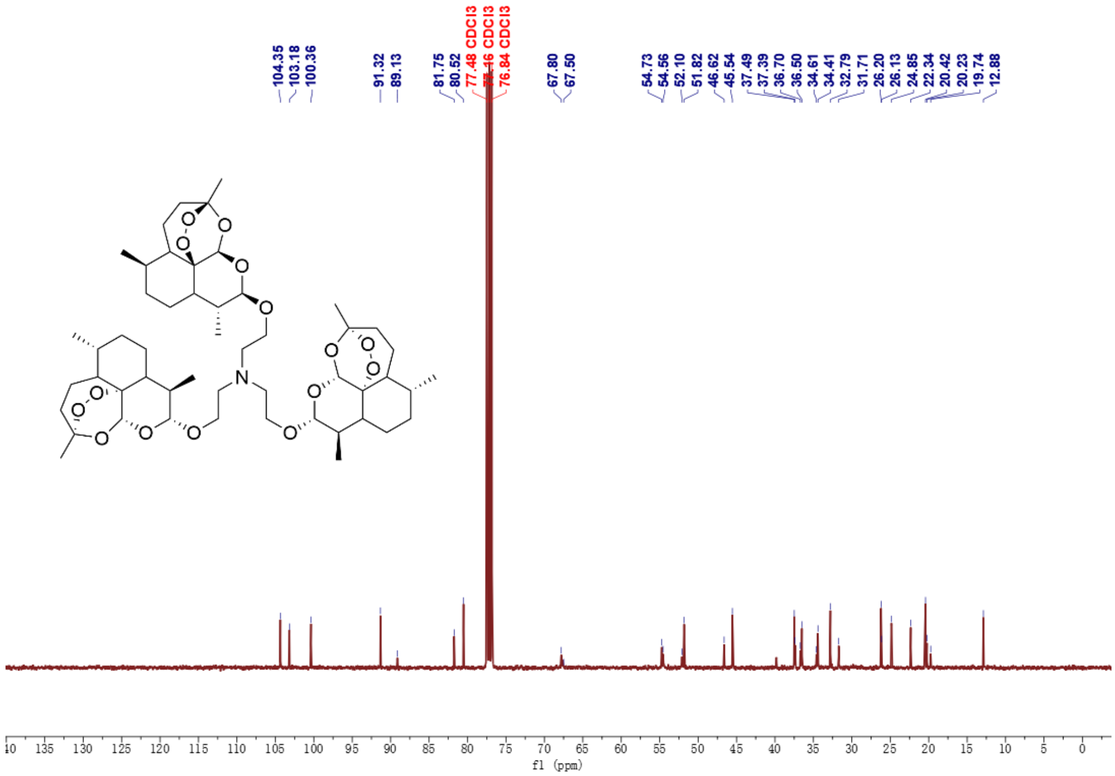

^{13}C NMR spectral data of compound **6d** (*α, α, α*)

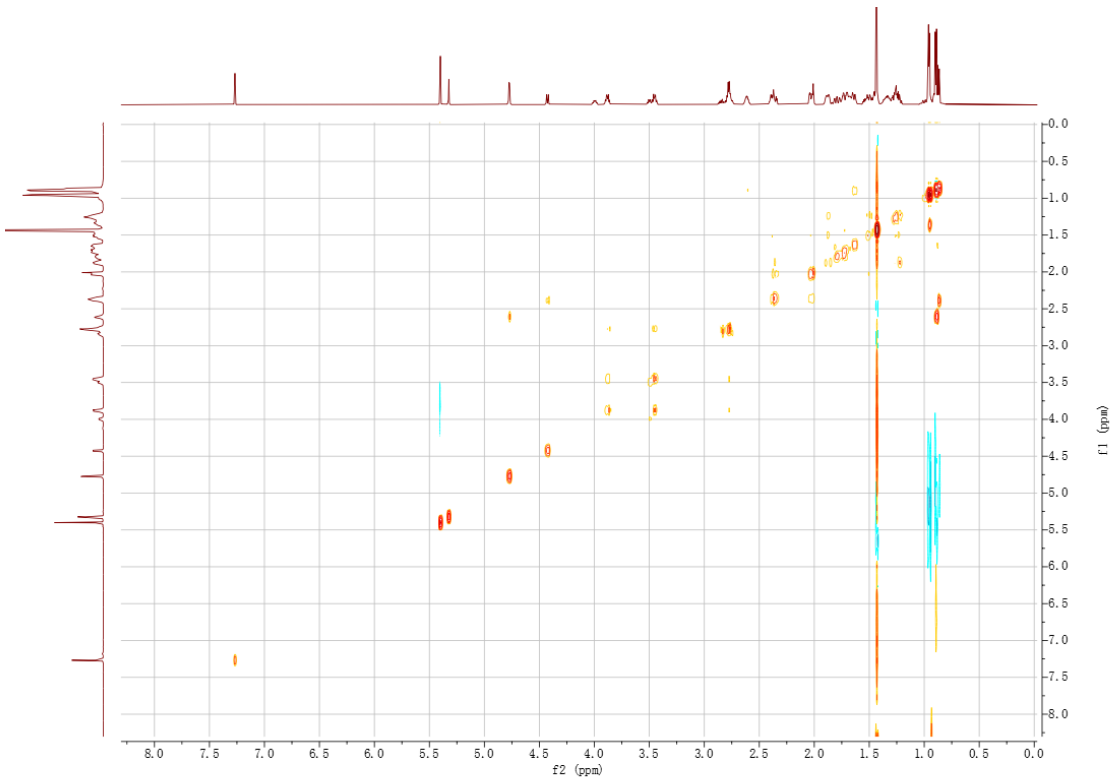

1H-1H COSY spectral data of compound **6b**

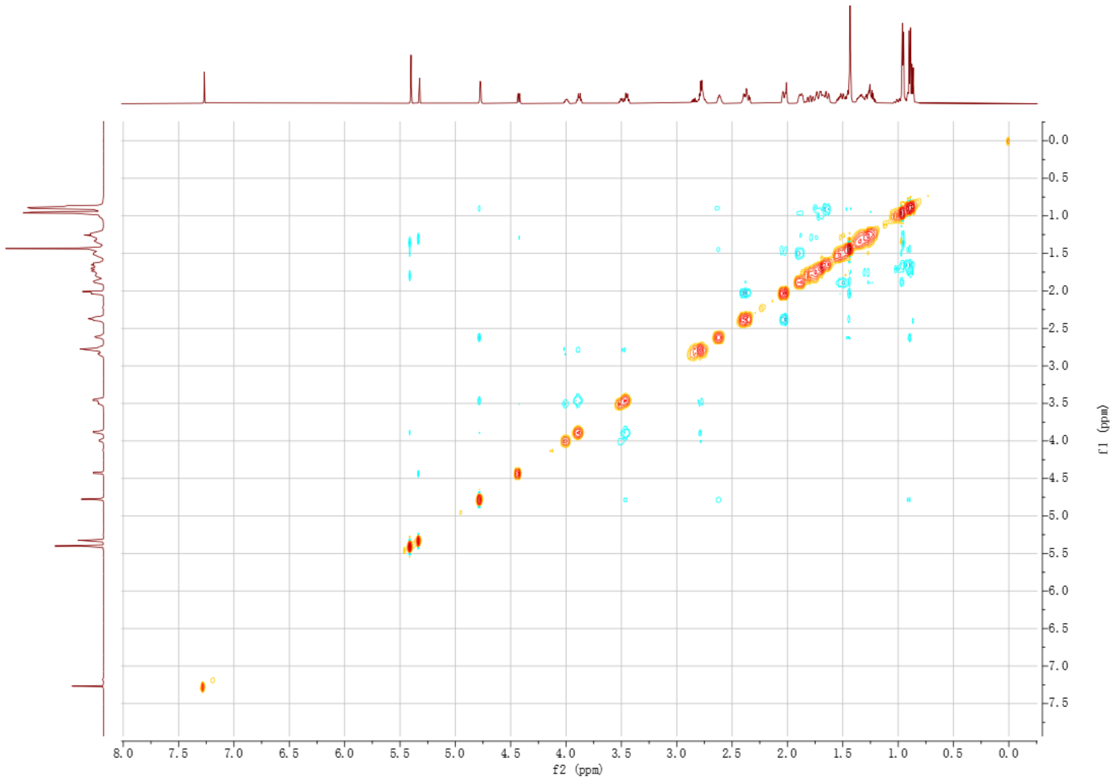

1H-1H NOESY spectral data of compound **6b**

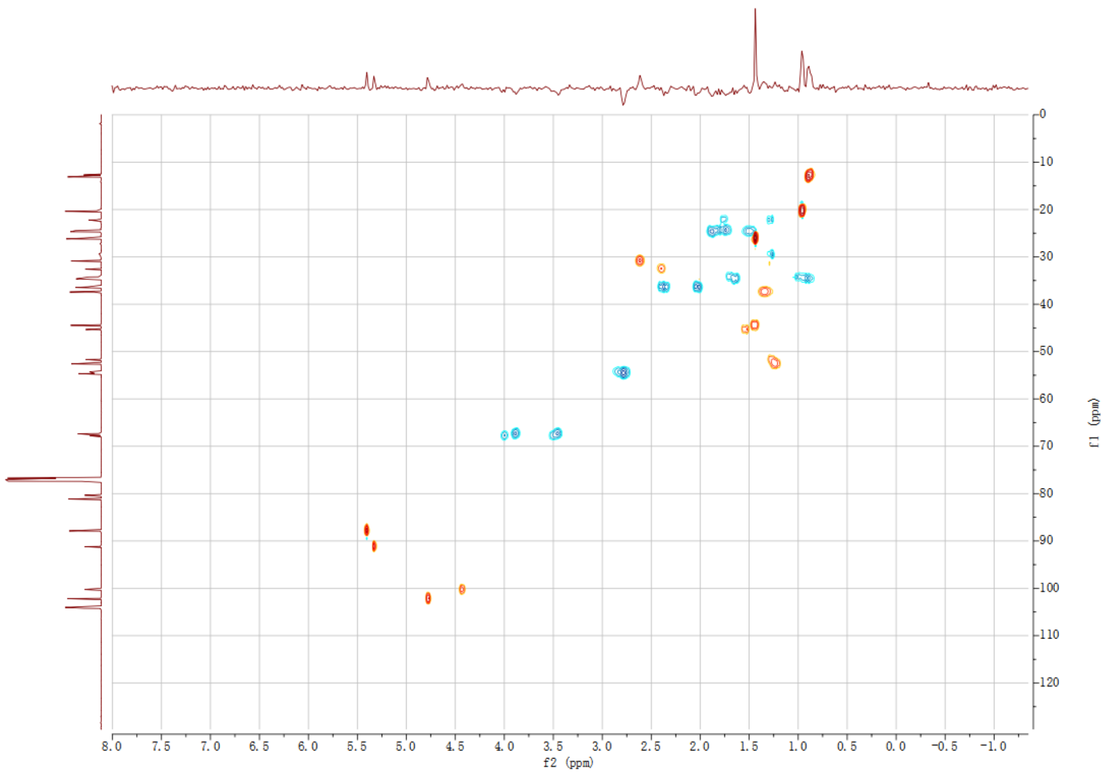

HMQC spectral data of compound **6b**

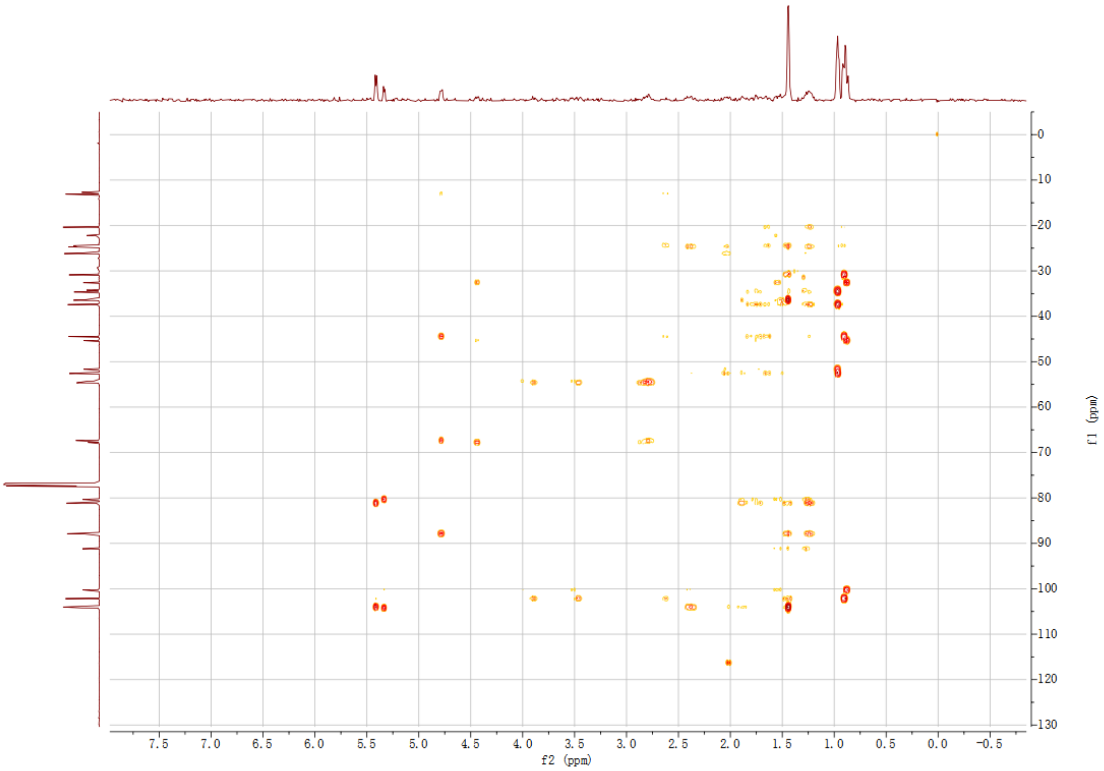

HMBC spectral data of compound **6b**

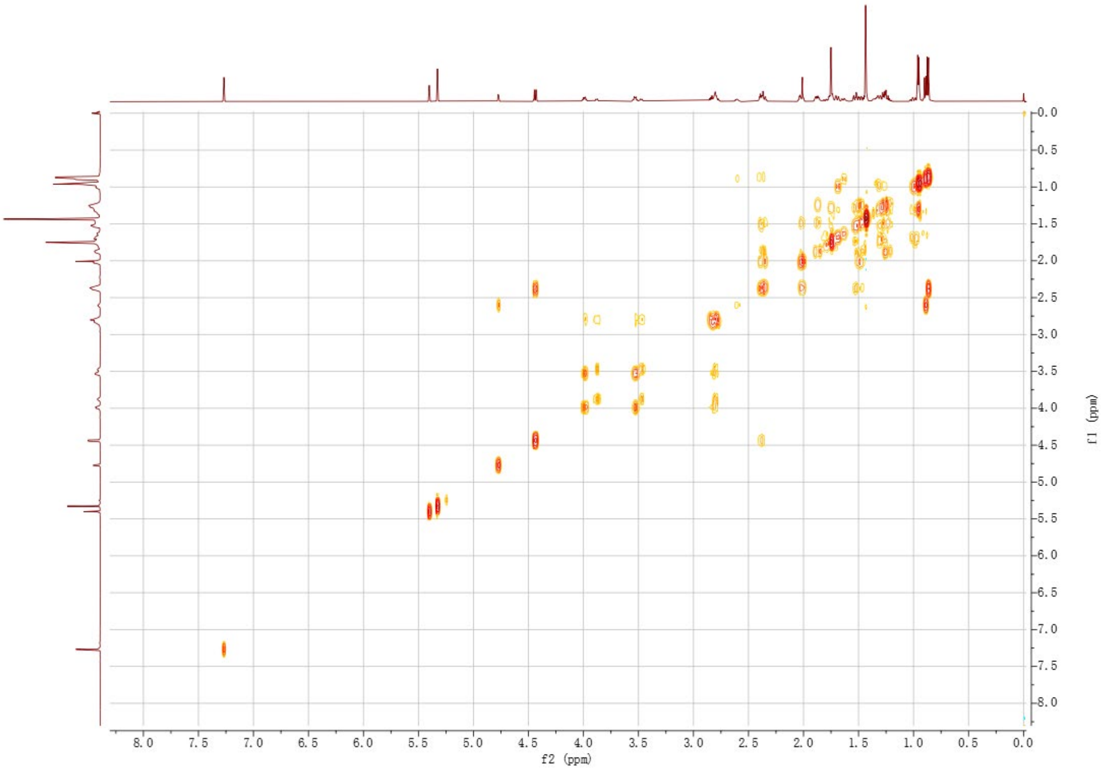

1H-1H COSY spectral data of compound **6c**

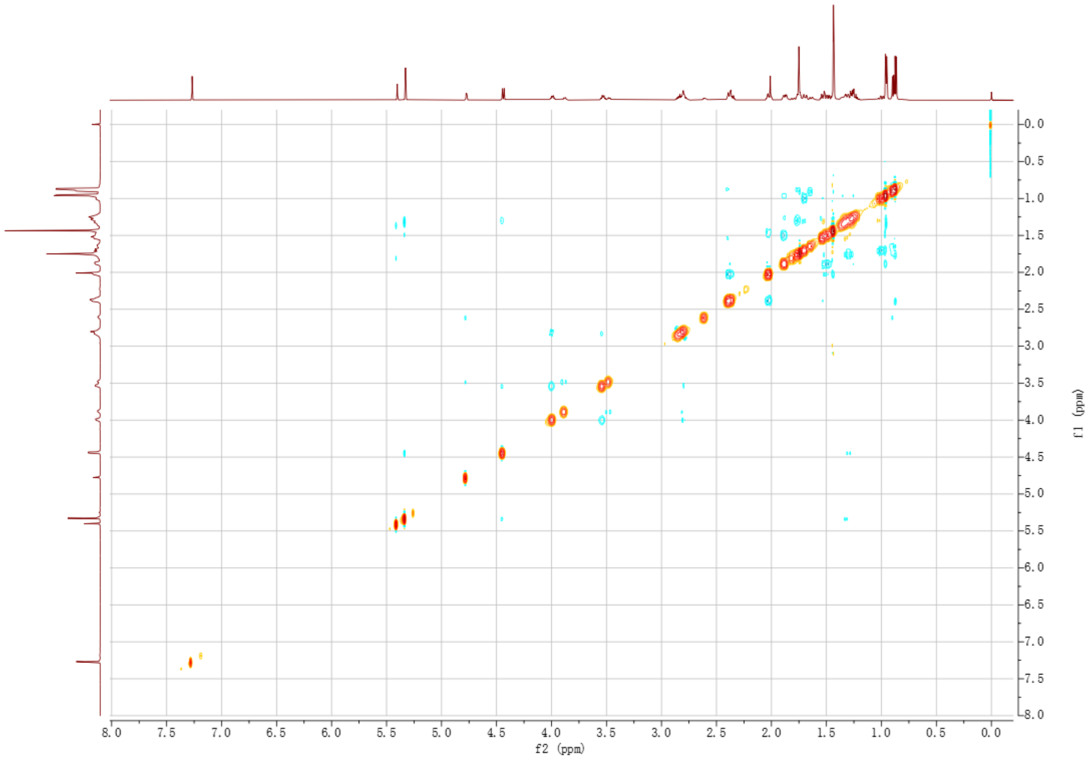

1H-1H NOESY spectral data of compound **6c**

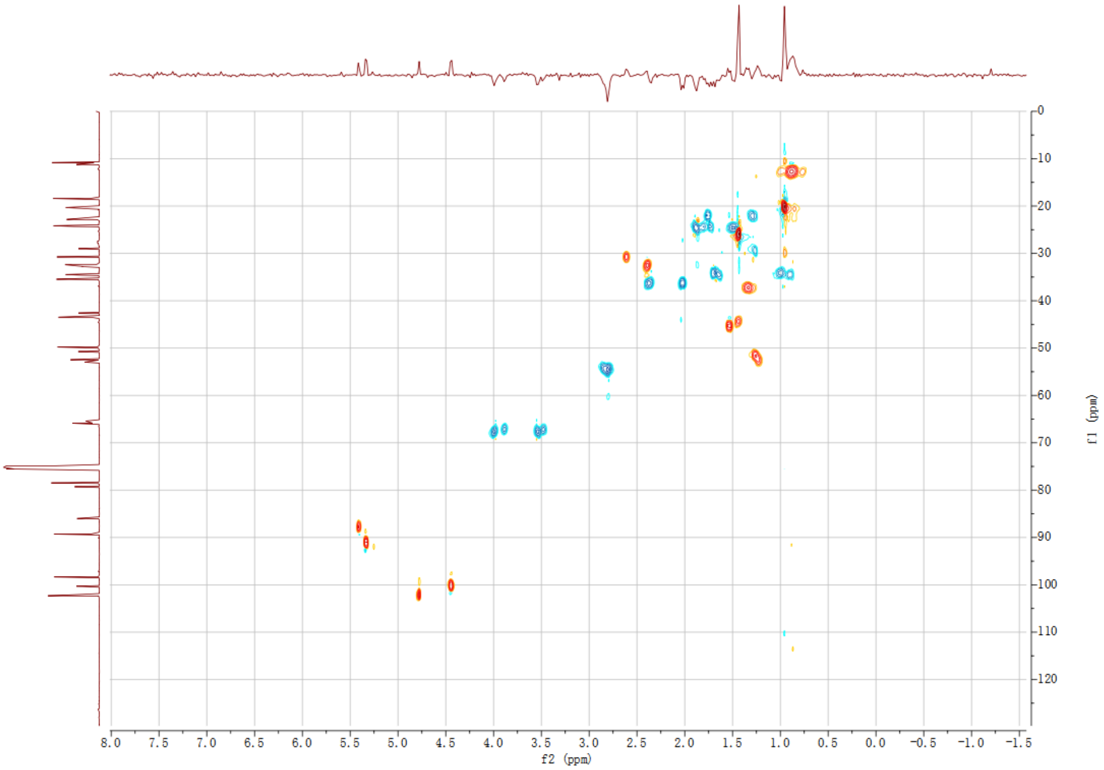

HMQC spectral data of compound **6c**

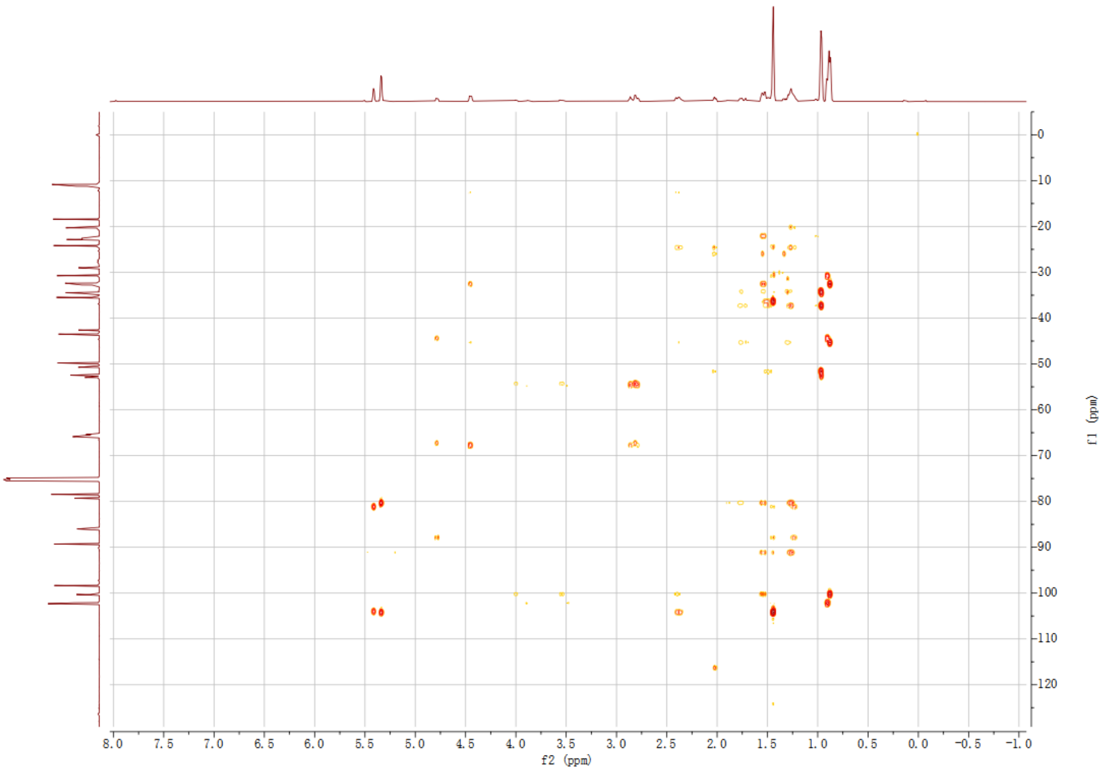

HMBC spectral data of compound **6c**

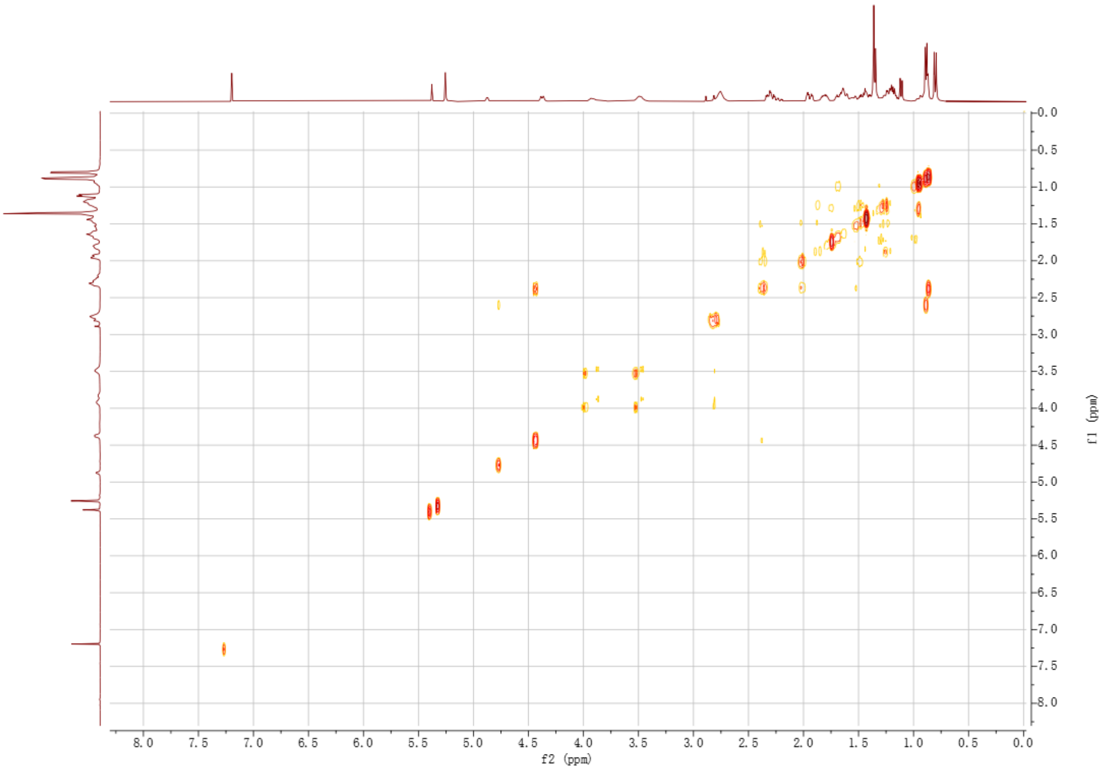

1H-1H COSY spectral data of compound **6d**

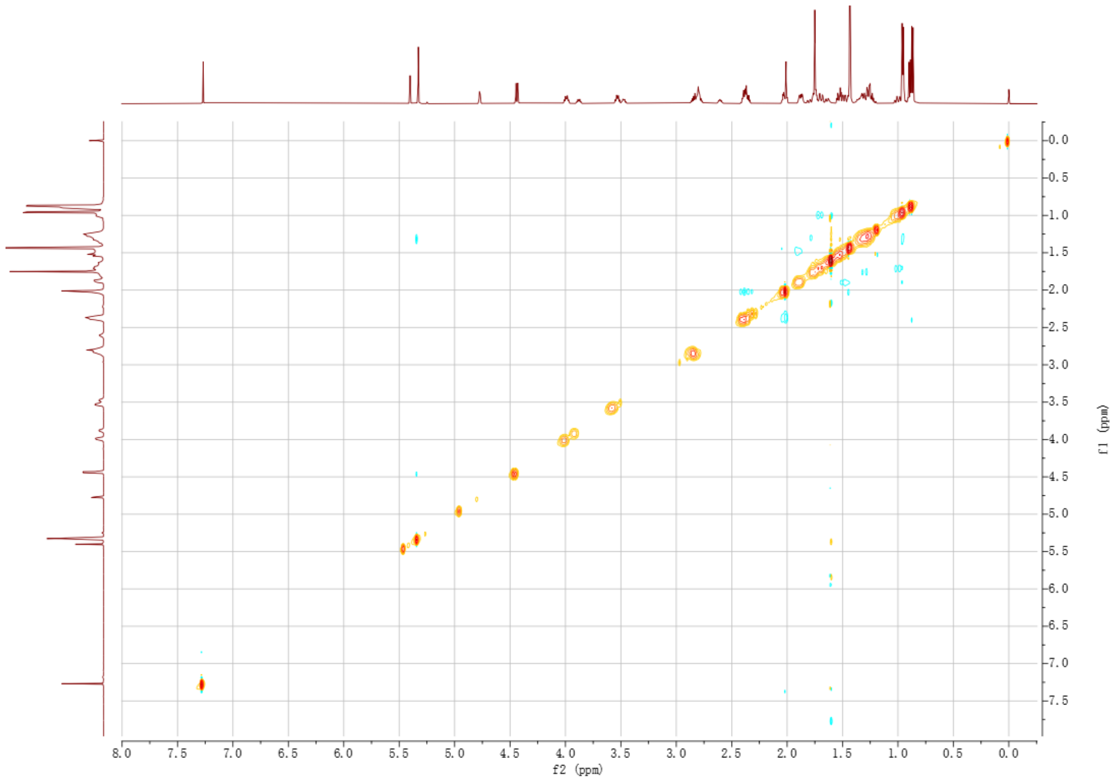

1H-1H NOESY spectral data of compound **6d**

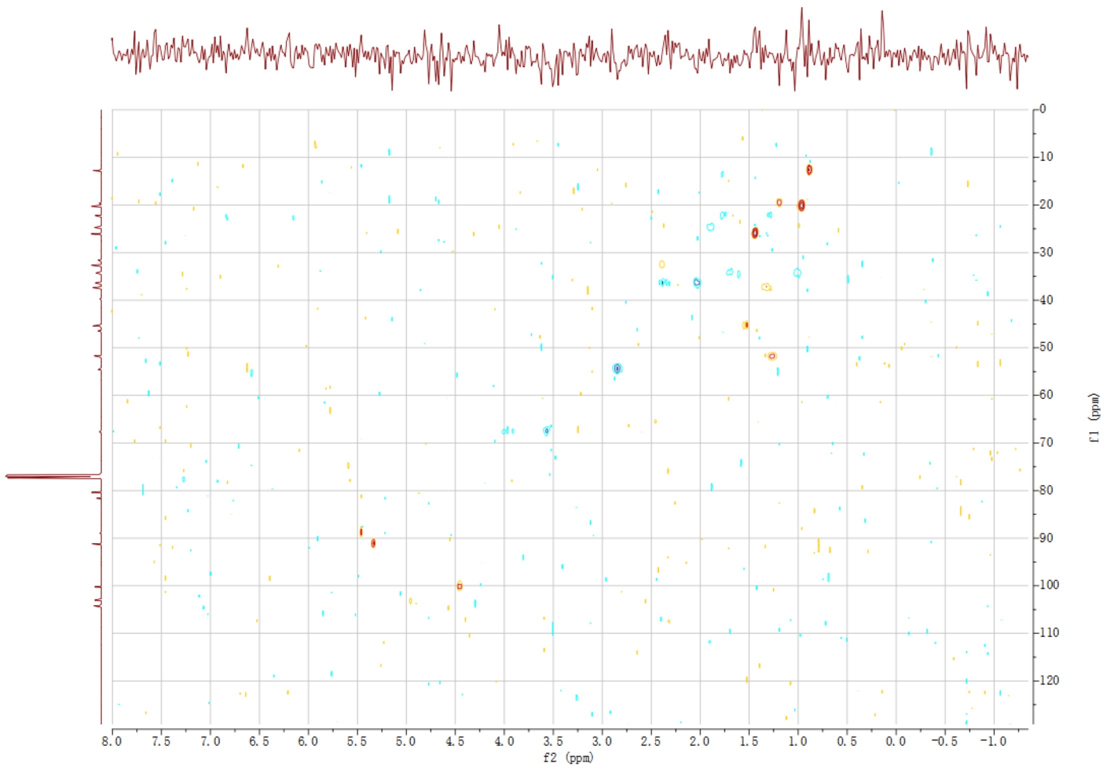

HMQC spectral data of compound **6d**

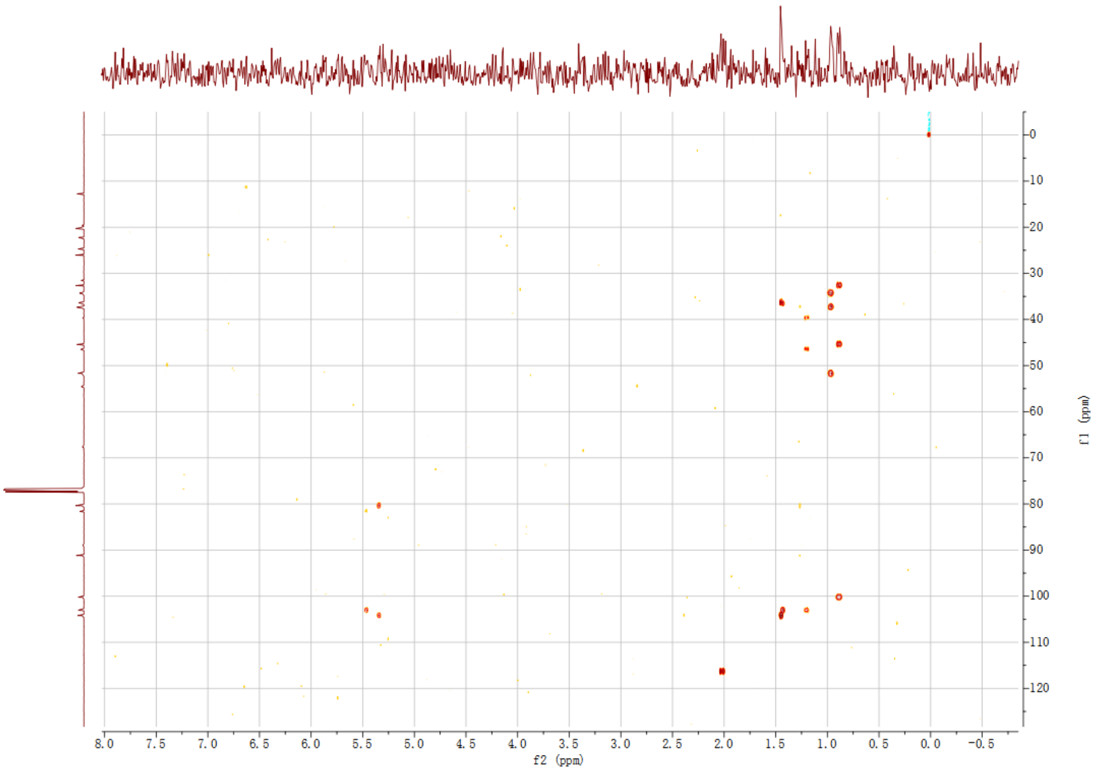

HMBC spectral data of compound **6d**
